# Condensins regulate resection–dependent DNA double–strand break repair pathways in replicated chromatin

**DOI:** 10.1093/nar/gkag076

**Published:** 2026-02-09

**Authors:** Mei Liu, Wei You, Lisa-Marie Weber, Emil Mladenov, Xixi Lin, Veronika Mladenova, Ramtin Omid Shafaat, Gabriel E Pantelias, Eleni Gkika, Martin Stuschke, Aashish Soni, George Iliakis

**Affiliations:** Division of Experimental Radiation Biology, Department of Radiation Therapy, University Hospital Essen, University of Duisburg-Essen, 45147 Essen, Germany; Institute of Medical Radiation Biology, University Hospital Essen, University of Duisburg-Essen, 45147 Essen, Germany; Division of Experimental Radiation Biology, Department of Radiation Therapy, University Hospital Essen, University of Duisburg-Essen, 45147 Essen, Germany; Institute of Medical Radiation Biology, University Hospital Essen, University of Duisburg-Essen, 45147 Essen, Germany; Division of Experimental Radiation Biology, Department of Radiation Therapy, University Hospital Essen, University of Duisburg-Essen, 45147 Essen, Germany; Institute of Medical Radiation Biology, University Hospital Essen, University of Duisburg-Essen, 45147 Essen, Germany; Division of Experimental Radiation Biology, Department of Radiation Therapy, University Hospital Essen, University of Duisburg-Essen, 45147 Essen, Germany; Institute of Medical Radiation Biology, University Hospital Essen, University of Duisburg-Essen, 45147 Essen, Germany; Division of Experimental Radiation Biology, Department of Radiation Therapy, University Hospital Essen, University of Duisburg-Essen, 45147 Essen, Germany; Institute of Medical Radiation Biology, University Hospital Essen, University of Duisburg-Essen, 45147 Essen, Germany; Division of Experimental Radiation Biology, Department of Radiation Therapy, University Hospital Essen, University of Duisburg-Essen, 45147 Essen, Germany; Institute of Medical Radiation Biology, University Hospital Essen, University of Duisburg-Essen, 45147 Essen, Germany; Division of Experimental Radiation Biology, Department of Radiation Therapy, University Hospital Essen, University of Duisburg-Essen, 45147 Essen, Germany; Institute of Nuclear Technology and Radiation Protection, National Centre for Scientific Research “Demokritos,” Aghia Paraskevi Attikis, 15310 Athens, Greece; Radiation Biology Laboratory, Department of Radiotherapy and Radiation Oncology, University Hospital Bonn, 53127 Bonn, Germany; Division of Experimental Radiation Biology, Department of Radiation Therapy, University Hospital Essen, University of Duisburg-Essen, 45147 Essen, Germany; German Cancer Consortium (DKTK), Partner Site University Hospital Essen, German Cancer Research Center (DKFZ), 45147 Essen, Germany; Division of Experimental Radiation Biology, Department of Radiation Therapy, University Hospital Essen, University of Duisburg-Essen, 45147 Essen, Germany; Institute of Medical Radiation Biology, University Hospital Essen, University of Duisburg-Essen, 45147 Essen, Germany; Division of Experimental Radiation Biology, Department of Radiation Therapy, University Hospital Essen, University of Duisburg-Essen, 45147 Essen, Germany; Institute of Medical Radiation Biology, University Hospital Essen, University of Duisburg-Essen, 45147 Essen, Germany; Radiation Biology Laboratory, Department of Radiotherapy and Radiation Oncology, University Hospital Bonn, 53127 Bonn, Germany

## Abstract

Condensins are key regulators of chromosome architecture and have emerging functions in DNA repair that are understudied. Here, we show that combined depletion of Condensin I and II in cell lines of normal and tumor origin selectively impairs DNA double-strand break (DSB) repair and the checkpoint response (DDR) specifically in the G_2_ phase of the cell cycle, with no detectable effects in G_1_ or S phase. Condensin knockdown increased cellular radiosensitivity and delayed in G_2_-phase, but not in asynchronous cells, the resolution of γH2AX and 53BP1 foci, indicating G_2_-specific defects in DSB repair. Mechanistically, condensin loss suppressed DNA end-resection and resection-dependent repair pathways, including homologous recombination (HR), single-strand annealing (SSA), and alternative end-joining (alt-EJ), but failed to significantly alter classical non-homologous end-joining (c-NHEJ). Reduced RAD51 and RPA70 foci formation in G_2_ confirmed inhibition of HR and DNA end resection. The G_2_ checkpoint was also compromised. Cytogenetic analysis revealed inhibition of chromosome break repair and visible chromatin decondensation, suggesting that condensins function to maintain an appropriate chromatin state for efficient DSB repair in G_2_-phase. These results identify for the first time condensins as G_2_ phase–specific regulators of genome stability by fine-tuning HR and other resection-dependent DSB repair pathways.

## Graphical Abstract

**Graphical Abstract:**
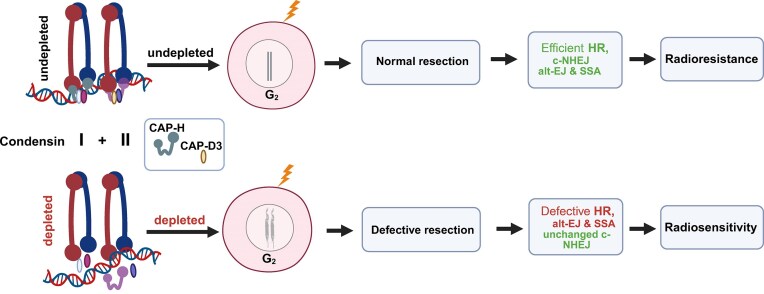

## Introduction

Condensins are essential regulators of chromatin architecture, required for chromosome condensation, genome organization, and gene regulation. Mammalian cells express two distinct Condensin complexes: Condensin I and Condensin II, each composed of the core SMC2 and SMC4 ATPases and unique non-SMC regulatory subunits [1–3]. Condensin I comprises of NCAPD2, NCAPG, and NCAPH, while Condensin II includes NCAPD3, NCAPG2, and NCAPH2. These complexes differ in subcellular localization -condensin I is cytoplasmic and gains DNA access after nuclear envelope breakdown, whereas Condensin II is nuclear throughout the cell cycle [4]. Both condensins participate in mitotic chromosome compaction by forming and enlarging DNA loops through loop extrusion [5–9]. Condensin I extrudes loops rapidly but transiently, while Condensin II extrudes loops more stably and contributes to larger-scale chromosomal structure [5].

Beyond mitosis, condensins also function in interphase nuclei, contributing to genome compartmentalization and transcriptional control [10–13]. Depletion of condensins causes chromatin decondensation [14, 15], underscoring their importance in maintaining higher-order chromatin structure. Evidence also suggests a role for condensins in DNA repair: Condensin II has been shown to promote homologous recombination (HR) [16, 17], while Condensin I may interact with PARP1 to support single-strand break repair in interphase [18, 19]. Additionally, Condensin I is excluded from under-replicated DNA in mitosis, potentially facilitating mitotic DNA synthesis [20]. Despite these insights, the precise role of condensins in DNA double-strand break (DSB) repair and the DNA damage response (DDR) remains only partially understood.

Ionizing radiation (IR) kills cells primarily by inducing DSBs, and the efficiency of their repair is a major determinant of the therapeutic success of radiotherapy (RT) [21, 22]. The main DSB repair pathways in mammalian cells include classical non homologous end joining (c-NHEJ) and HR. c-NHEJ operates throughout the cell cycle with fast kinetics and tolerates sequence loss or mis-joining [23]. In contrast, HR is largely error-free, uses the sister chromatid as a template, and is confined to the S and G_2_ phases [23–26]. Cells also engage alternative end joining (alt-EJ) and single-strand annealing (SSA), both of which are error-prone and can result in deletions or chromosomal rearrangements [24, 27–31].

HR, SSA, and alt-EJ share a common entry step: DNA end resection, catalyzed by the CtIP/MRN complex [27, 32–35]. This resection step is not required for c-NHEJ, and indeed it inhibits it. Thus, the choice of DSB repair pathway is tightly regulated and depends on both the cell cycle phase and chromatin context. Understanding the determinants of repair pathway choice is critical for the mechanistic understanding of RT effects. We previously demonstrated that although HR engages more than 50% of DSBs at low doses of radiation when the load of DSBs in the genome is low, its contribution rapidly decreases as the dose of radiation increases and is undetectable at IR doses above 10 Gy. This DSB-load-dependent confinement of HR is not mediated by the function of either 53BP1 or RAD52 [26]. We hypothesized, therefore, that chromatin decondensation, accompanying high radiation doses [36], could contribute to this suppression. Supporting this, we found that inducing chromatin decondensation with hypotonic treatment had minimal effects on c-NHEJ and alt-EJ but strongly inhibited HR while promoting SSA [37].

Given their role in chromatin compaction, we reasoned that condensins may help maintain the chromatin environment needed for HR engagement. We therefore investigated the contribution of condensins to DSB repair after IR. In this study, we show that combined depletion of Condensin I and II disrupts DSB repair selectively in G_2_-phase cells. Condensins knockdown has little effect on c-NHEJ, but suppresses resection-dependent pathways (HR, SSA, alt-EJ), impairs the G_2_ checkpoint, and induces visible chromatin decondensation. These findings suggest that condensins preserve genome stability by maintaining a chromatin state permissive for HR and other resection-dependent pathways in G_2_. The results identify condensins as potential targets for radiosensitization in the clinical application of radiation.

## Materials and methods

### Cell lines

RPE-1 and 82–6 hTert cells were cultured as a monolayer in DMEM growth medium supplemented with 10% fetal bovine serum (FBS), 100 µg/mL penicillin, and 100 µg/mL streptomycin. A549, U2OS DR-GFP, U2OS SA-GFP, U2OS EJ2-GFP, and U2OS EJ5-GFP cells were maintained in McCoy’s 5A medium supplemented with 10% FBS, 100 µg/mL penicillin, 100 µg/mL streptomycin, and 2 µg/mL puromycin. All cell lines were maintained in a 95% air, 5% CO₂ atmosphere at 37°C and were routinely tested for mycoplasma contamination.

### X-ray irradiation

Cells were irradiated at room temperature (RT) using an X-ray generator (GE Healthcare, Buckinghamshire, UK) set at 320 kV and 10 mA. Distances were adjusted based on dish size to ensure even dose distribution over the dishes. Dose rates were 3.6 Gy/min at 50 cm and 1.7 Gy/min at 75 cm distance from the anode. For G_2_/M checkpoint assays, cells were irradiated on a warm plate (42°C water bath) at 66 cm distance, with a dose rate of 2.2 Gy/min; this was necessary to maintain the temperature in the cells at approximately 37°C, as temperature shocks generate G_2_ arrest of similar size to radiation. Control samples were sham-irradiated.

### Inhibitors

The ATM inhibitor KU-55933 and ATR inhibitor VE-821 (Haoyuan Chemexpress) were used at 10 µM and 5 µM final concentration. DNA2 inhibitor (NIH, Developmental Therapeutics Program, Cat# NSC-105808) was used at a final concentration of 4 µM. Mirin, an MRE11 inhibitor (2-amino-5-[(4-hydroxyphenyl) methylene]-4(5H)-thiazolone, Santa Cruz Biotechnology) was used at 75 µM final concentration. All inhibitors were dissolved in dimethylsulfoxide (DMSO) and administered 1 h before irradiation, while control cells were treated with equivalent amounts of DMSO. After irradiation, cells were promptly returned to the incubator, and inhibitors were maintained throughout the experimental procedures.

### Gene knockdown

Specific siRNAs (see Supplementary Table S1) targeting CAP-H, CAP-D3, and SMC2 were delivered using the Amaxa Nucleofector 2D device (Lonza) or Gene Pulser X electroporation apparatus (Bio-Rad). The X-20 program was used for A549 cells, and the X-01 program for the U2OS cell group. RPE-1 cells were transfected using the Gene Pulser X. Knockdown efficiency was assessed 48 h post-transfection via Western blot analysis.

### Clonogenic survival assay

Cells transfected with control siRNA, siCAP-H, siCAP-D3, or their combination were irradiated 48 h post-transfection. Immediately after irradiation, cells were trypsinized, seeded at low densities, and incubated at 37°C to form colonies. After 11 days for RPE-1 cells and 14 days for 82–6 hTert, U2OS, and A549 cells, colonies were fixed and stained with crystal violet. Colonies were counted to calculate plating efficiency and survival fractions.

### DSB repair reporter systems

U2OS GFP reporter cell lines [38] were used to quantify repair of I-SceI-induced DSBs via specific repair pathways. The DR-GFP construct analyzed HR; EJ5-GFP, NHEJ (total or distal end-joining); SA-GFP, SSA; and EJ2-GFP, alt-EJ [26]. Reporters are stably integrated into the genome. Indicated cell lines were re-transfected with the I-SceI expression plasmid pCMV-3xNLS-ISceI (1 µg/10^6^ cells) and respective siRNAs 48 h after the initial siRNA transfection. Knockdown efficiency was verified by Western blotting. GFP expression was measured by flow cytometry (Gallios, Beckman Coulter) at 48 h post I-SceI transfection. Repair efficiency was calculated as the fraction of GFP-positive cells.

### G_2_ premature chromosome condensation (PCC)

The previously reported protocol was followed [39]. Briefly, 0.2–0.6 × 10^6^ cells were plated in 25 cm² flasks post-siRNA transfection. After two days, cells were irradiated with 0.5 Gy X-rays. Calyculin A (50 nM) was added 45 min before harvesting to induce PCC. This incubation was included in the repair time. After treatment, cells were harvested, centrifuged at 1200 rpm for 5 min at 4°C, and incubated in hypotonic solution (75 mM KCl) for 10 min at RT. Carnoy’s fixative was used for fixation. The cell suspension was dropped onto wet slides and air-dried overnight. Slides were stained with 3% Giemsa in Sorenson’s buffer for 10 min, rinsed with tap water, and air-dried. Finally, slides were mounted with Entellan (Merck-Millipore). Approximately 100 G_2_-PCCs were scored per time point. Gaps were only considered when larger than the chromatid width. Scoring was done under a bright-field microscope (Olympus VANOX-T, Japan).

### Metaphase preparation

Exponentially growing cells were irradiated with 1 Gy X-rays and harvested 1 or 4 h post-IR. Colcemid was added during the last hour for the 4 h point and at 15 min post-IR for 45 min for the 1 h point. Cells were subjected to 10 min hypotonic treatment at RT and fixed three times in Carnoy’s fixative. Metaphase spreads were stained with 3% Giemsa. Fifty metaphases per time point were scored for chromatid breaks (gaps counted only if wider than chromatid width) using bright-field microscopy (Olympus VANOX-T, Japan). M-FISH was performed as previously described [40, 41].

### Protein extraction

Cells were harvested by trypsinization, resuspended in PBS, and centrifuged. Cell pellets were lysed in RIPA buffer (Thermo Scientific) with protease inhibitors (1000:10 ratio). 3 × 10^6^ cells were lysed in 100 μL RIPA. Lysates were shaken at 4°C for 15 min, sonicated, and then centrifuged at 13 000 rpm for 15 min at 4°C to collect protein extracts.

### SDS-PAGE and western blot analysis

Protein concentration was determined by the Bradford assay. Sixty µg of protein were mixed 1:1 with 2 × loading buffer, heated at 96°C for 5 min, and centrifuged. Samples were resolved on 10% or 4–15% SDS-PAGE (Mini-PROTEAN TGX, Bio-Rad) at 120 V for 120 min. Proteins were transferred onto nitrocellulose membranes (Odyssey® 0.22 μm) at 280 mA for 2 h at 4°C. Membranes were blocked in 5% non-fat milk in TBS for 1 h, incubated overnight with primary antibodies (Supplementary Table S2) in TBS-T with milk, washed, then incubated with secondary antibodies (Supplementary Table S2). Detection was done with the Odyssey® Infrared Imaging System (LI-COR Biosciences).

### Cell cycle analysis

Cells were fixed in cold 70% ethanol and stored at 4°C. Before flow cytometry, cells were centrifuged, ethanol removed, and resuspended in PI + RNase solution, and incubated at 37°C for 15 min. DNA content was analyzed using a Gallios flow cytometer (Beckman Coulter). Cell cycle phases were quantified using Wincycle software.

### Determination of mitotic index

A two-parameter FACS protocol [42, 43] using phospho-Histone H3 (H3pS10) staining was employed. ∼1 × 10⁵ cells were plated, grown for 48 h, and treated with inhibitors or DMSO 1 h prior to IR. Cells were irradiated on a warm plate and collected in prewarmed medium, fixed in cold 70% EtOH and stored at –20°C. Cells were permeabilized with 0.25% Triton X-100 on ice, blocked with PBG, stained with primary and secondary antibodies (Supplementary Table S2), then with PI/RNase. Samples were measured on a Gallios flow cytometer (Beckman Coulter) and analyzed with Kaluza software (Beckman Coulter).

### Cell sorting

Cell sorting was done on an Astrios cell sorter (Beckman Coulter). Prior to cell sorting, cells were transfected with either a non-coding siRNA (siNC) or siRNAs specifically targeting CAP-D3 and CAP-H (siCAP-D3 and siCAP-H) and cultured for 48 h. Efficient knockdown of the targeted proteins was confirmed by Western blot analysis. For sorting in defined cell-cycle phases, cells were directly stained for 1 h *in vitro* with Hoechst 33342 (5 μg/mL). This staining condition allows clear discrimination of cell-cycle phases without compromising cell viability. After staining, cells were harvested by trypsinization and resuspended at 2 × 10^6^ cells/mL in pre-warmed growth medium supplemented with the same concentration of Hoechst 33342. Cell populations were first characterized based on forward and side scatter (FSC/SSC), and single cells were selected using the 405–546/20 height versus 405–546/20 width parameters. Cells in G_1_, S, and G_2_ phases were sorted using gates defined on the 405–488/59 area channel. Sorting efficiency and purity were assessed by re-analyzing the collected G_1_, S, and G_2_ cell fractions.

### RT-qPCR

Sorted knockdown and control cells were processed for mRNA and small RNA isolation using the miRNeasy Kit™ (QIAGEN) according to the manufacturer’s protocol, and purified small RNAs were stored at − 80°C. For RT-qPCR, 1 µg of total RNA was reverse-transcribed using the High-Capacity cDNA Reverse Transcription Kit (Applied Biosystems) in a 20 µL reaction. Gene expression of Condensin subunits (NCAPH, NCAPD3), and resection genes (MRE11, CtIP, BLM, DNA2, EXO1) was assessed using TaqMan™ assays. RT-qPCR reactions contained 5 µL of 2 × TaqMan™ Fast Advanced Master Mix, 0.5 µL of each 20 × assay, and 2 µL water, dispensed into 384-well plates at 7.5 µL per well, followed by the addition of 2.5 µL of 1:10 diluted cDNA. All reactions were run in technical triplicates across two biological replicates, with NTC and RT-negative controls included. Amplification was performed on a QuantStudio 7 Pro system under standard cycling conditions (95°C for 20 s, then 40 cycles of 95°C for 1 s and 60°C for 20 s). Relative expression was normalized to ACTB (beta-actin) and calculated using the 2^-ΔCt method. Gene names and the assay IDs used in RT-qPCR are listed in Table S5. The corresponding Minimum Information for Publication of Quantitative Real-Time PCR Experiments (MIQE) checklist is provided in the supplementary materials (Table S6).

### Immunofluorescence staining

Performed as described before [26, 44]. Cells grown on poly-L-lysine-coated coverslips were pulse-labeled with 10 µM EdU for 30 min before IR. The pulse labeling of S-phase cells using EdU facilitates the cell cycle-specific analysis of repair foci and allows discrimination between the foci kinetics in G_1_, S, and G_2_ phases of the cell cycle. For RPA70 staining, cells were permeabilized in PBS with 0.25% Triton X-100 for 5 min on ice; this step was omitted for γ-H2AX, 53BP1, and RAD51 staining. After PBS wash, cells were fixed in 3% paraformaldehyde/2% sucrose for 15 min at RT, permeabilized in P-solution for 10 min at RT, blocked in PBG overnight at 4°C, and incubated with primary antibodies (Supplementary Table S2) for 2 h at RT. Secondary antibodies (AF568 or AF647) were applied for 1.5 h, followed by Click-iT EdU cocktail, DAPI counterstaining (200 ng/mL), and mounting in ProLong Gold Antifade (Thermo Fisher). Slides were scanned using an AxioScan.Z1 (Zeiss). Quantitative image-based cytometry (QIBC) was used for analysis [26]. Foci were scored in cell cycle-specific populations using Imaris and Orange software. Foci values obtained at 0 Gy were subtracted before data visualization. The corresponding baseline measurements are presented in Supplementary Tables S3 and S4.

### Pulsed-field gel electrophoresis (PFGE)

PFGE was performed to measure DSB induction and repair as previously described [45]. DNA fragmentation was quantified by calculating the fraction of DNA released (FDR). EtBr-stained gels were imaged with a Typhoon Imager and analyzed with ImageQuant 5.2 (GE Healthcare). Dose-response curves (FDR vs. IR dose) were used to derive dose equivalents (DEQ). Repair kinetics were plotted as DEQ vs. time. Mean ± SD was calculated from six replicates across two independent experiments.

### Statistical analysis

Most results represent the mean ± SD from two to three independent experiments. Data analysis and plotting were performed using SigmaPlot 14. Non-linear regression (SigmaPlot 14) was used for colony formation assays. Significance was determined using the Student’s t-test (SigmaPlot 14). Statistical significance is indicated as: *P < 0.05, **P < 0.01, ***P < 0.001, ****P < 0.0001.

## Results

### Suppression of condensin expression sensitizes cells to IR

**Figure 1.**
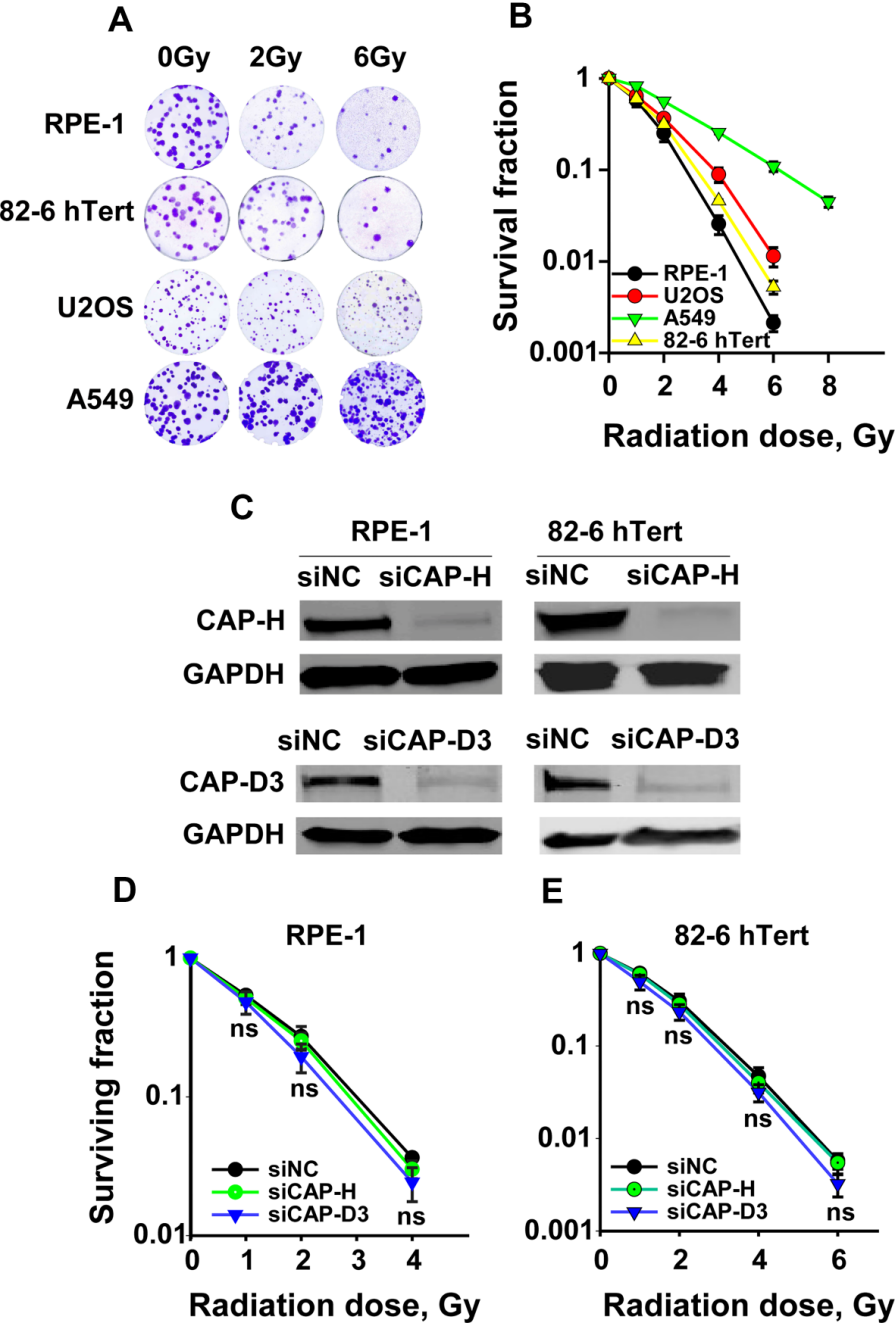
Depletion of condensin I or condensin II alone fails to induce radiosensitization. **(A)** Representative colony formation dishes at selected IR doses in the indicated cell lines; **(B)** Clonogenic survival curves of RPE-1, 82–6 hTert, U2OS, and A549 cell lines exposed to a spectrum of IR doses. **(C)** Western blot analysis of single siCAP-H or single siCAP-D3 depletion at 48 h post-transfection using specific siRNA; GAPDH served as loading control; **(D)** Clonogenic survival assay of RPE-1 cells upon single depletion of siCAP-H or siCAP-D3; **(E)** Same as in panel **(D)**, but for 82–6 hTert cells. Clonogenic survival data represent means ± SD from three independent experiments.

We began our investigation by assessing the impact of Condensin I and Condensin II knockdown on the radiosensitivity of human epithelial (RPE-1), fibroblast (82–6hTert), lung carcinoma (A549), and osteosarcoma (U2OS) cell lines. Baseline radiosensitivity of these cell lines is shown in Fig. 1A and B. To selectively inhibit Condensin I or II, we targeted CAP-H and CAP-D3, specific subunits of Condensin I and II, respectively (see Introduction). CAP-D3 was targeted using a previously validated oligo [15] in RPE-1 and 82–6 hTert cells. Four different siRNA oligonucleotides were screened for the knockdown of CAP-H and SMC2. A cocktail of all four siRNAs was found to be most efficient for the knockdown of CAP-H (Supplementary Fig. S1A). A combination of siRNAs #1 and #4 was chosen for SMC2 knockdown (Supplementary Fig. S1B). Cell cycle distribution remained unaffected by individual Condensin knockdown in both RPE-1 and 82–6 hTert cells (Supplementary Fig. S1C–F). Despite efficient suppression of CAP-H and CAP-D3 levels (Fig. 1C), individual knockdowns failed to sensitize cells to radiation (Fig. 1D–E).

**Figure 2.**
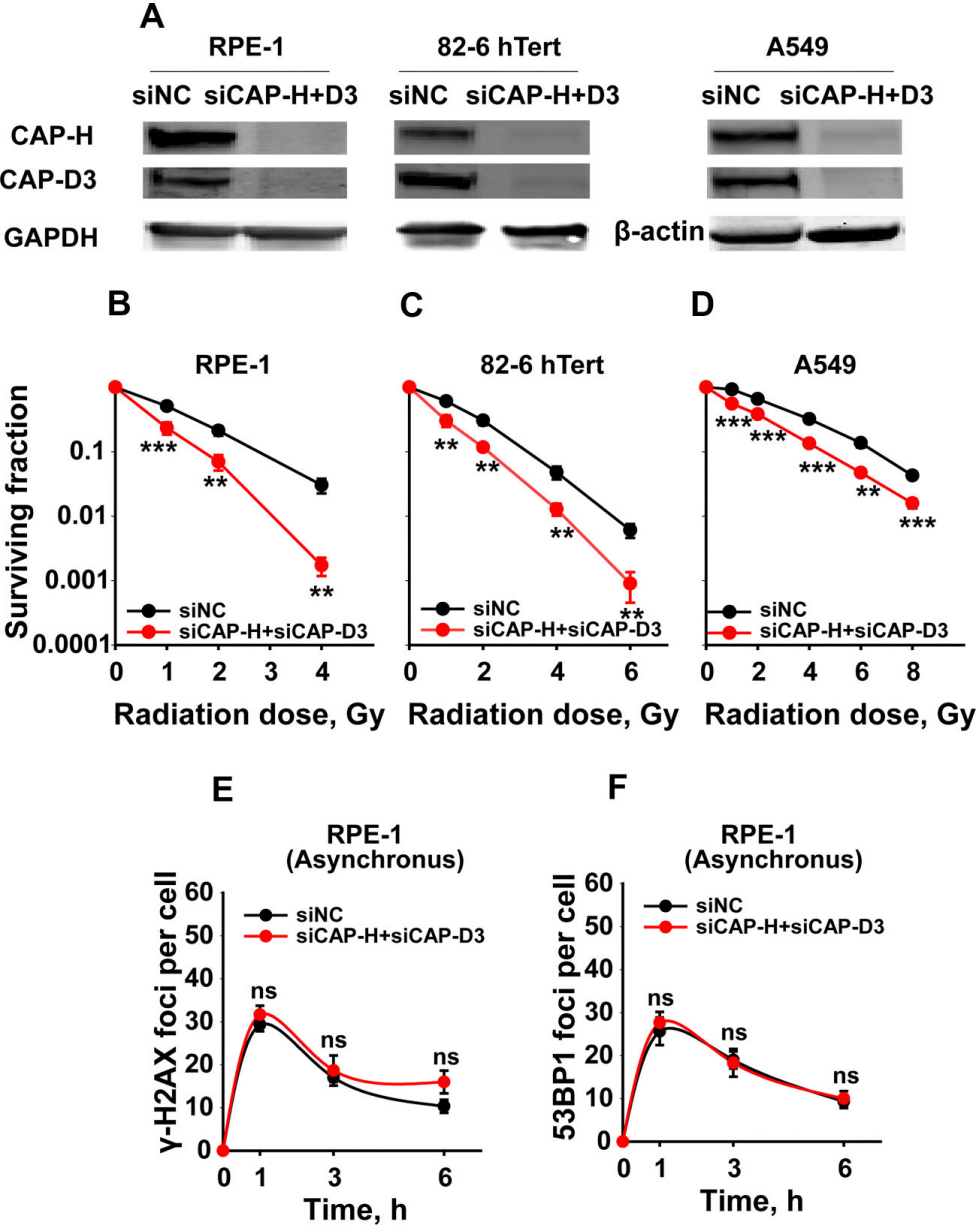
Combined knockdown of condensins I and II robustly radiosensitizes human cells and impairs DSB repair in the G_2_ phase. **(A)** Western blot analysis to detect CAP-H and CAP-D3 protein levels in RPE-1, 82–6 hTert, and A549 cells 48 h post-transfection, after combined depletion of CAP-H and CAP-D3; GAPDH served as loading control; **(B)** Clonogenic survival assay of RPE-1 cells upon combined depletion of siCAP-H and siCAP-D3. **(C)** Same as in panel **(B)**, but for 82–6 hTert cells. **(D)** Same as in panel **(B)**, but for A549 cells. **(E)** Kinetics of γH2AX foci in RPE-1 asynchronous cells depleted from condensins. **(F)** Kinetics of 53BP1 foci in RPE-1 asynchronous cells depleted of condensins. Data represent the mean ± SD from three independent experiments.

We considered functional redundancy between Condensin complexes and, therefore, examined the effect of simultaneous CAP-H and CAP-D3 knockdown in RPE-1, 82–6 hTert, and A549 cells (Fig. 2A). Strikingly, combined knockdown resulted in a robust radiosensitization in all three cell lines (Fig. 2B–D), without significantly altering the cell cycle (Supplementary Fig. S2A–F). This result points to overlapping/redundant roles of Condensin I and II in cell radiosensitivity.

We also assessed the impact of SMC2 knockdown, one of the two core subunits common to both Condensin complexes. Although SMC2 expression was efficiently suppressed in RPE-1 cells, 82–6 hTert cells, and A549 cells (Supplementary Fig. S3A–C), this knockdown caused severe cytotoxicity in unirradiated cells (Supplementary Fig. S3D–F) and generated aberrant mitoses with chromosomes showing clear signs of decondensation (Supplementary Fig. S3G), precluding reliable analysis of radiation effects. SMC2 depletion also altered cell cycle profiles, increasing G_1_ and decreasing S-phase populations (Supplementary Figs. S4A–F), although these effects were not as pronounced as observed in cytotoxicity and on the chromosome level. Based on these findings, subsequent experiments were conducted using exclusively, combined knockdown of CAP-H and CAP-D3, hereafter referred to as “condensins suppression.” Furthermore, since all cell lines tested respond similarly, in the following sections, we mainly present results with RPE-1 cells and show, under Supplementary Materials, relevant results with A549 cells.

### Condensins suppression impairs DSB repair only in the G_2_ phase of the cell cycle

We investigated whether the radiosensitization observed upon condensin suppression derives from defective DSB repair. Therefore, we measured next the kinetics of γH2AX and 53BP1 foci formation and decay after 1 Gy of IR. Notably, in asynchronously growing RPE-1 cells, condensin suppression had no measurable effect on γH2AX or 53BP1 foci kinetics (Fig. 2E and F). Similar results were also obtained in A549 cells (Supplementary Fig. S5 A–D). Given the rather specialized functions of condensins in G_2_/M to mediate chromosome condensation and segregation, we inquired whether their effects on DSB repair also have a cell cycle component.

**Figure 3.**
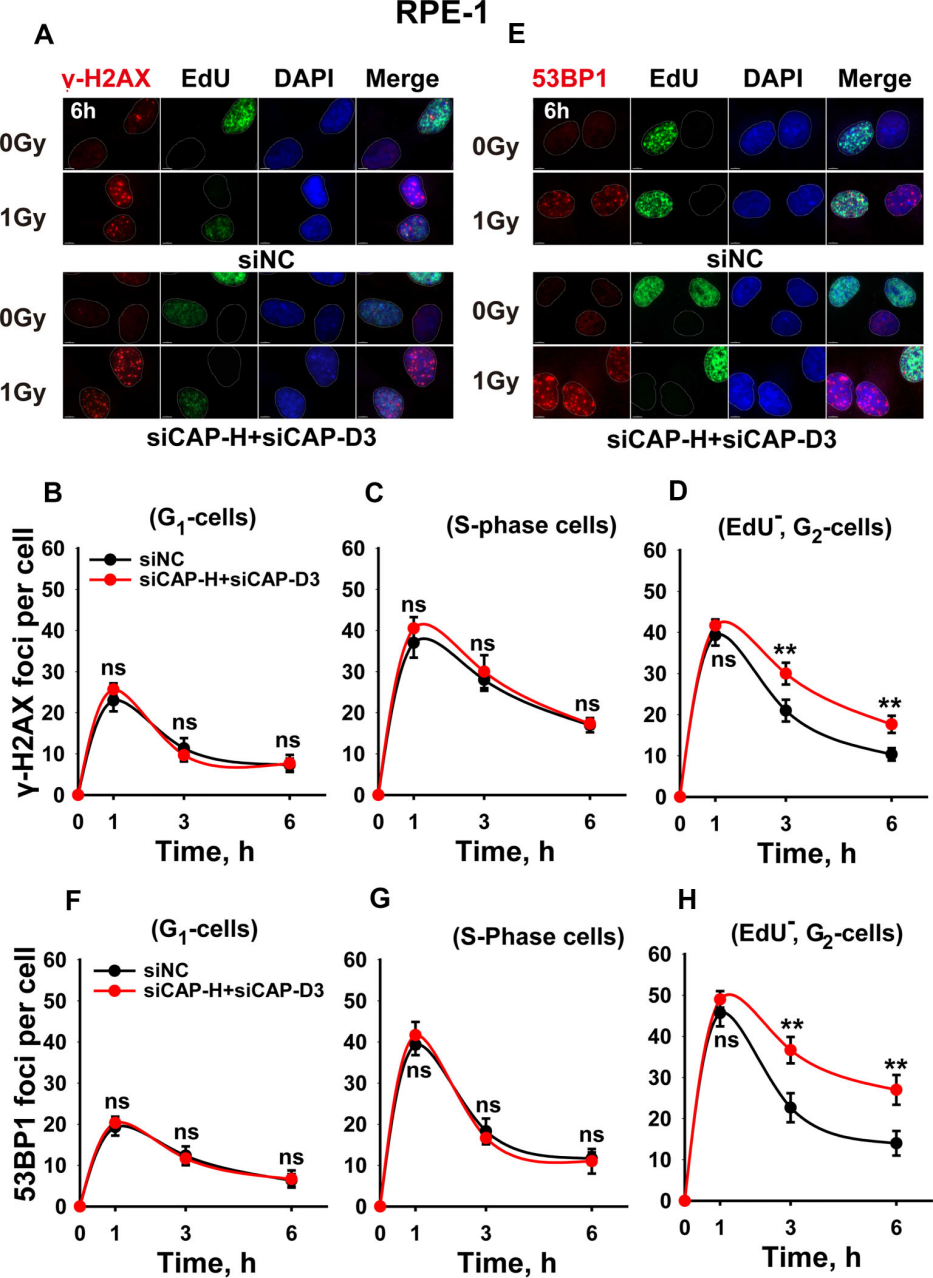
Cell cycle-specific impact of condensin depletion on the repair of IR-induced DSBs in RPE-1 cells. **(A)** Representative images of γH2AX foci in RPE-1 cells irradiated with 1 Gy X-rays and collected at the indicated post-IR times; **(B)** Kinetics of γH2AX foci in EdU^−^, G_1_-phase cells depleted from condensins. **(C)** Kinetics of γH2AX foci in EdU^+^, S-phase cells depleted from condensins. **(D)** G_2_-specific quantification of γ-H2AX foci upon combined depletion of siCAP-H + siCAP-D3. **(E)** Representative images of 53BP1 foci upon 1 Gy irradiation. **(F)** Kinetics of 53BP1 foci in EdU^−^, G_1_-phase cells depleted of condensins. **(G)** Kinetics of 53BP1 foci in EdU^+^, S-phase cells depleted of condensins. **(H)** G_2_-specific quantification of 53BP1 foci upon depletion of condensins. Data represent the mean ± SD from three independent experiments.

We therefore utilized QIBC for sound statistics and analyzed γH2AX and 53BP1 kinetics after exposure to 1 Gy, separately in G_1_-, S-, and G_2_-phase of the cell cycle (S6A). For this analysis, it was necessary to include a 30-min exposure to EdU just before irradiation – to label cells undergoing DNA replication – and combine it with DAPI staining for cell cycle identification. Cells in G_1_ or G_2_ phase of the cell cycle could now be identified by the DAPI signal and confirmed as non-S-phase by being EdU^-^. On the other hand, EdU ^+^ cells were unequivocally classified as S-phase cells at the time of irradiation (Supplementary Fig. S6A). Analysis of RPE-1 cells in this way shows that condensin suppression does not affect the kinetics of γH2AX in G_1_– or S-phase (Fig. 3A–C). Strikingly, however, cells analyzed in G_2_-phase show a marked inhibition of DSB repair (Fig. 3D). This result is also mirrored in the analysis of 53BP1 foci kinetics (Fig. 3E–H), as well as by a parallel set of experiments in A549 cells (Supplementary Fig. S6B-G). We conclude that condensin suppression selectively impairs DSB repair in G_2_-phase cells.

### Condensin knockdown inhibits HR in the G_2_ phase

**Figure 4.**
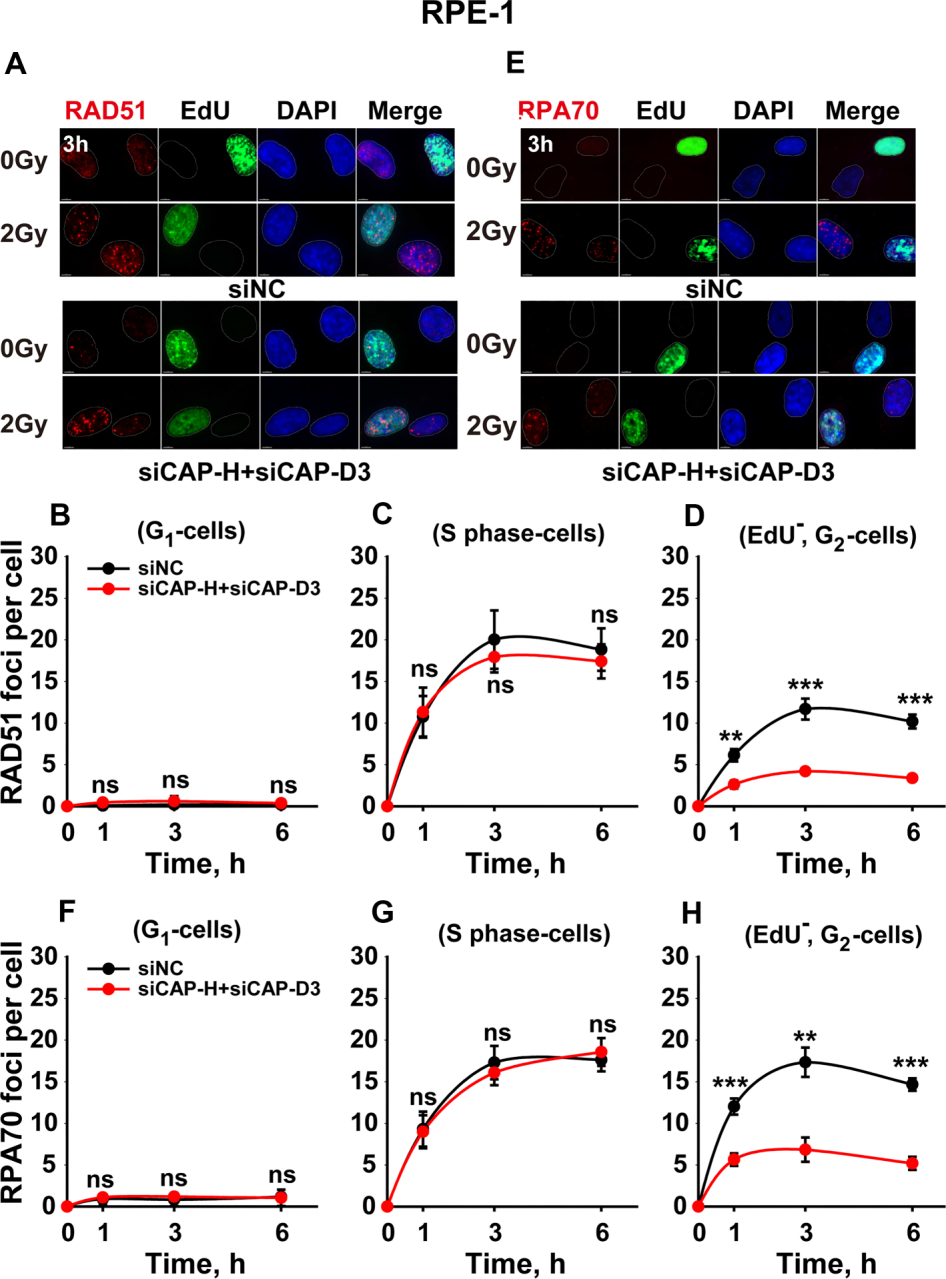
Impact of condensin depletion on the kinetics of RAD51 foci and RPA70 foci in RPE-1 cells. **(A)** Representative images of Rad51 foci in cells irradiated with 2 Gy of X-rays and collected at the indicated time after irradiation; **(B)** Kinetics of Rad51 foci in EdU^−^, G_1_-phase cells depleted of both condensins. **(C)** Kinetics of Rad51 foci in EdU^+^, S-phase cells depleted of both condensins. **(D)** G_2_-specific quantification of Rad51 foci upon combined depletion of condensins. **(E)** Representative images of RPA70 foci irradiated with 2 Gy of X-rays. **(F)** Kinetics of RPA70 foci in EdU^−^, G_1_-phase cells depleted of both condensins. **(G)** Kinetics of RPA70 foci in EdU^+^, S-phase cells depleted of both condensins. **(H)** G_2_-specific quantification of RPA70 foci upon combined depletion of condensins. Data represent the mean ± SD from three independent experiments.

The above results raised the question as to whether the G2-specific effect reflects inhibition of HR, which is fully active in the G_2_-phase. We analyzed, therefore, RAD51 foci formation and decay (Fig. 4A–D). RAD51, a central HR protein, accumulates at resected DNA ends to promote strand invasion. In RPE-1 cells, RAD51 foci were as expected at background levels in G_1_-phase (Fig. 4B). There is clear RAD51 foci development in S-phase, which remains unaffected by condensin knockdown (Fig. 4C). Notably, however, analysis in the G_2_-phase shows pronounced inhibition of RAD51 foci development, reflecting a strong inhibition of HR (Fig. 4D). Similar results are obtained in A549 cells (Supplementary Fig. S7A–D). Along with the suppression of HR, also DNA end-resection is similarly affected, specifically in G_2_-phase RPE-1 cells (Fig. 4 E–H), a result that is also confirmed in A549 cells (Supplementary Fig. S8A–D). Thus, condensin knockdown suppresses resection and HR, but only in the G_2_-phase of the cell cycle.

### Reporter assays confirm that condensins suppress resection-dependent DSB-repair

**Figure 5.**
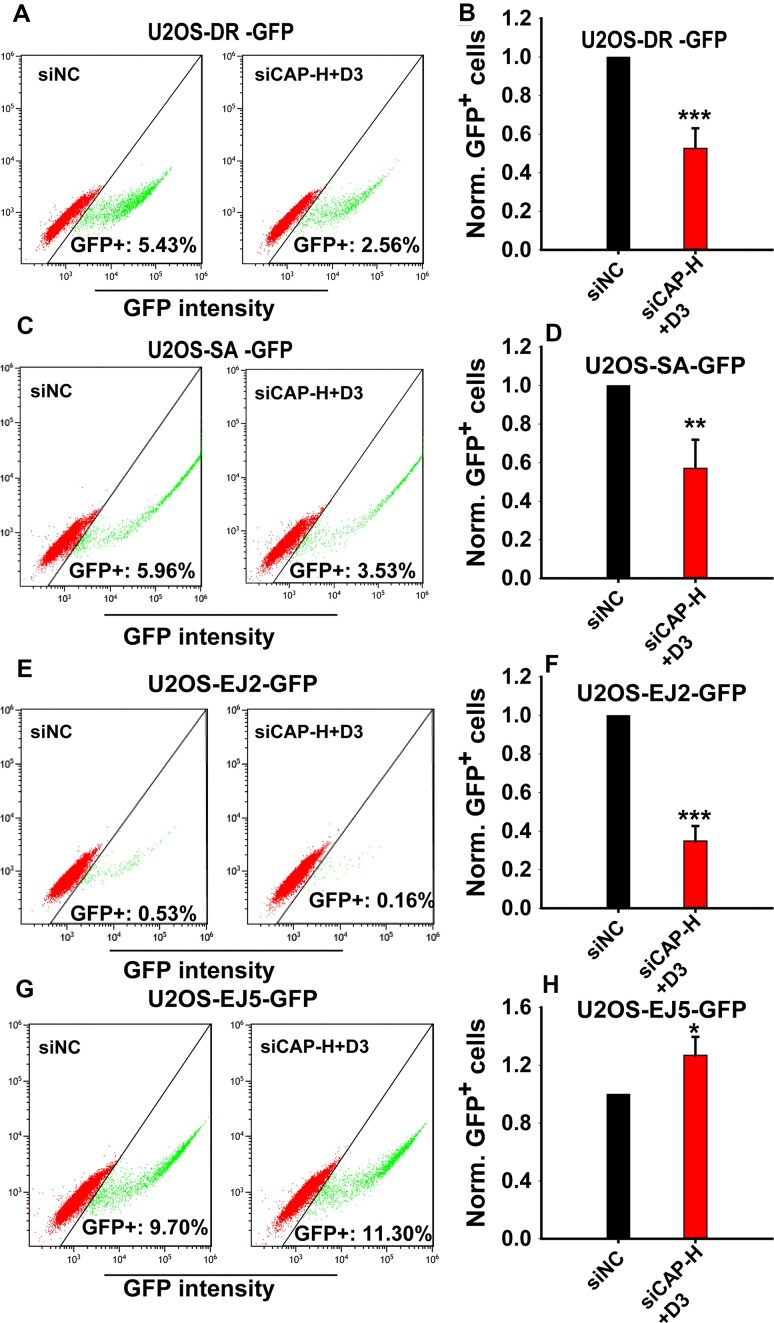
Impact of condensin depletion on HR, SSA, alt-EJ, and NHEJ in reporter U2OS cells. The widely used GFP-reporter U2OS cells, specifically designed to report the repair of I-SceI-induced DSBs by HR (DR-GFP), SSA (SA-GFP), alt-EJ (EJ2-GFP), and NHEJ (EJ5-GFP), were employed. **(A)** Percentage of GFP-positive cells, in siNC or CAP-H + CAP-D3-depleted, DR-GFP cells. **(B)** Frequency of HR upon combined depletion of CAP-H + CAP-D3. (**C** and **D**) Same as panels (A and B), but for SA-GFP cells. (**E** and **F**) Same as panels (A and B), but for EJ2-GFP cells. (**G** and **H**) Same as panels (A–C), but for EJ5-GFP cells. Data represent the mean ± SD from three independent experiments.

To explore the functions of condensins in DSB repair using alternative approaches, we employed a battery of U2OS reporter cell lines that monitor pathway-specific DSB repair of an I-SceI-induced DSB. Cells with the DR-GFP construct report effects on HR and show ∼50% suppression in HR efficiency following suppression of condensin expression (Supplementary Fig. S9A, Fig. 5A and B). Although the effect is less pronounced than in RAD51 foci development and decay, it should be noted that larger effects would be anticipated if cell cycle-specific analysis were possible in this kind of experiment as well.

Although HR suppression is often accompanied by a compensatory increase of SSA [37, 46], analysis of the corresponding reporter cell line U2OS-SA-GPF shows instead ∼ 50% reduction (Supplementary Fig. S9B, Fig. 5C and D). A marked effect (∼70%) is also observed with the U2OS EJ2-GFP reporter cell line that detects a specific class of alt-EJ effects (Supplementary Fig. S9C, Fig. 5E and F). Notably, U2OS-EJ5-GFP cells that also detect NHEJ events show no inhibition in the corresponding events (Supplementary Fig. S9D, 5G and H). These results collectively show an effect of condensins on resection-dependent DSB repair.

To confirm with independent methods the contribution of condensins to DSB repair by c-NHEJ, we employed PFGE. Supplementary Fig. S9E summarizes the results obtained with exponentially growing A549 cells and shows that knockdown of condensins has no detectable effect on c-NHEJ. Even after inhibition of DNA-PKcs to evaluate the effect on alt-EJ, contributions of condensins remained undetectable. We conclude that condensin suppression leaves end-joining pathways largely unchanged. We attribute the quantitative differences observed in the effects of reporter assays and IF analysis or PFGE to the lack of cell cycle-specific analysis in the former and the high doses of IR utilized for PFGE. We have reported that high loads of DSBs suppress resection-dependent DSB repair pathways [26, 39].

### The suppression of DSB repair in G_2_- but not S-phase, after condensins knockdown does not derive from altered expression of components of the resection apparatus

To get insights into the mechanistic foundations of the G_2_-specificity of the condensin effect, we designed experiments to test whether it derives from cell cycle-dependent fluctuations in the abundance of key components of the resection machinery. This inquiry requires specific analysis of cells in the G1, S and G2 phases of the cell cycle. Because standard synchronization methods fail to provide cell populations with the required sharpness in S and G_2_ phase, we opted for cell sorting, despite the associated difficulty of obtaining large numbers of cells. Supplementary Fig. S10 shows an experiment carried out with A549 cells after condensin knockdown. Supplementary Fig. S10A confirms the efficiency of the knockdown in this experiment, whereas Supplementary Fig. S10B and C show the cell cycle distributions of the starting populations and the gates applied. The lower panels show the cell cycle distribution of the sorted populations. It is evident that satisfactory enrichment in G_1_, S, and G2 phases could be achieved.

**Figure 6.**
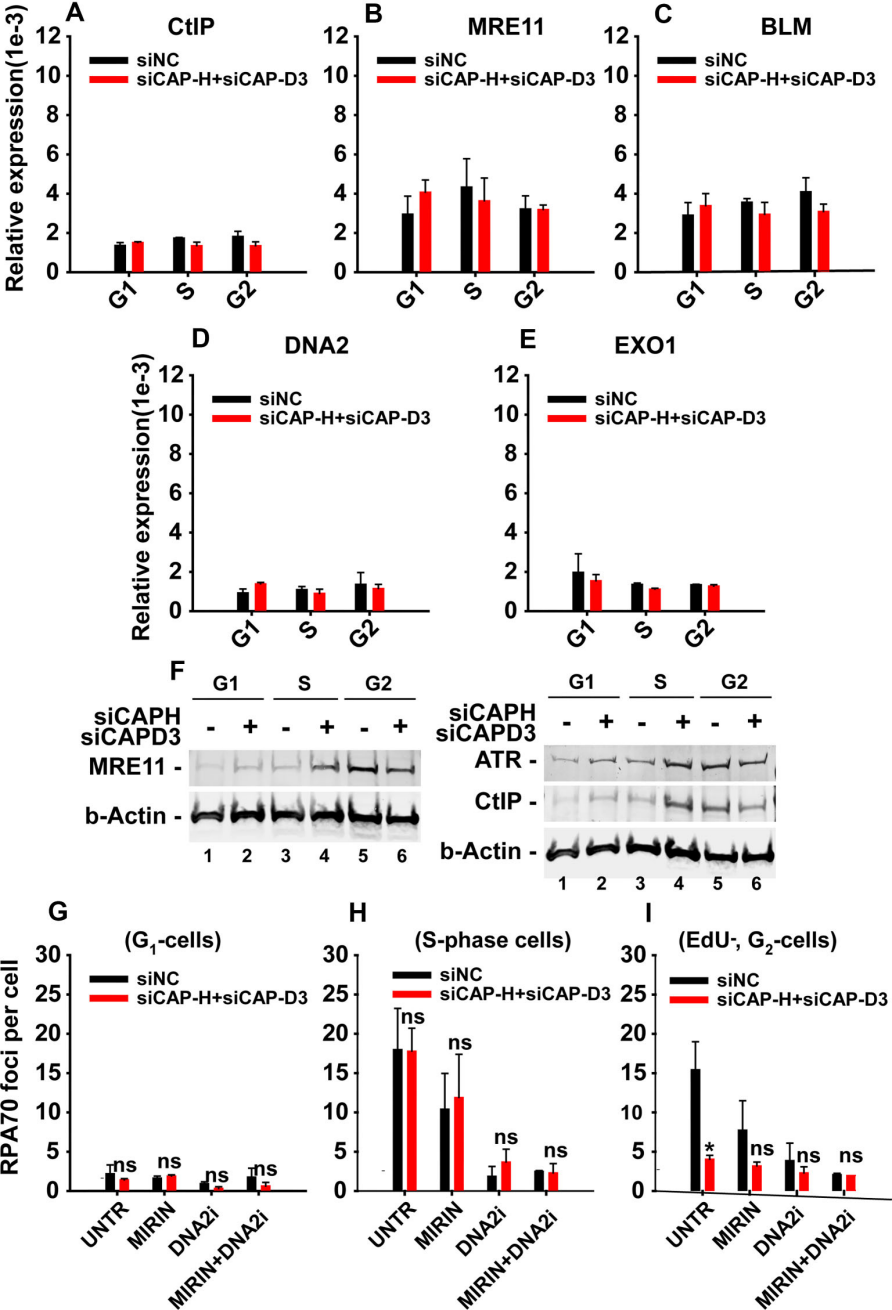
Impact of condensin knockdown on cell cycle-specific expression of resection proteins in sorted G_1_, S, and G_2_ A549 cells. The relative expression of indicated resection proteins was measured using RT-qPCR and Western blotting. Additionally, the impact of chemical inhibition of MRE11 and DNA2 was assessed on the degree of DSB end resection in irradiated A549 cells in a cell cycle-specific manner. **(A)** Relative expression of CtIP measured by RT-qPCR upon condensin depletion in sorted G_1_, S, and G_2_ cells. **(B)** Same as in panel **(A)**, but for MRE11 expression. **(C)** As in panel **(A)**, but for BLM expression. **(D)** As in panel **(A)**, but for DNA2 expression. **(E)** As in panel **(A)**, but for EXO1 expression. **(F)** Expression of MRE11 and CtIP upon condensin depletion was measured by Western blot in sorted G_1_, S, and G_2_ cells; beta-actin served as a loading control. **(G)** Impact of chemical inhibition of MRE11 and DNA2 alone and in combination on the degree of DSB end resection measured by scoring RPA70 foci in irradiated G_1_ cells. **(H)** As in panel **(G)**, but in irradiated S-phase cells. **(I)** As in panel **(G)**, but in irradiated G_2_-phase cells. RT-qPCR and RPA70 foci data represent the mean ± SD from two independent experiments.

We used RT-qPCR analyses to investigate fluctuations in the expression of CtIP, MRE11, BLM, DNA2, or Exo1 in condensins-depleted, sorted G_1_-, S-, and G_2_-phase cells. The results in Supplementary Fig. S11A and B confirm at the mRNA level the efficient knockdown of the individual condensins, CAP-H and CAP-D3. The analysis of populations in the different phases of the cell cycle did not reveal significant changes in the expression of CtIP, MRE11, BLM, DNA2, and EXO1 proteins (Fig. 6A–E, respectively), indicating that the G_2_-specific effects on DSB repair are not caused by altered expression of resection proteins. Western blot analysis of sorted and irradiated G_1_-, S-, and G_2_-phase cells also fails to show any changes in the expression of MRE11 and CtIP in condensin-depleted cells (Fig. 6F). Additionally, inhibiting MRE11 or DNA2 with specific inhibitors had no effect in G_1_ cells, and produced no further impact than the condensins knockdown alone in S- and G_2_ cells (Fig. 6G–I, respectively). Collectively, these results indicate that the core resection machinery remains in itself functional throughout the cell cycle and that activity changes cannot be invoked to explain the reduced resection observed specifically in G2 phase after condensin knockdown.

### Condensins suppression has only a minor effect on the activation and recovery of the G_2_ checkpoint

**Figure 7.**
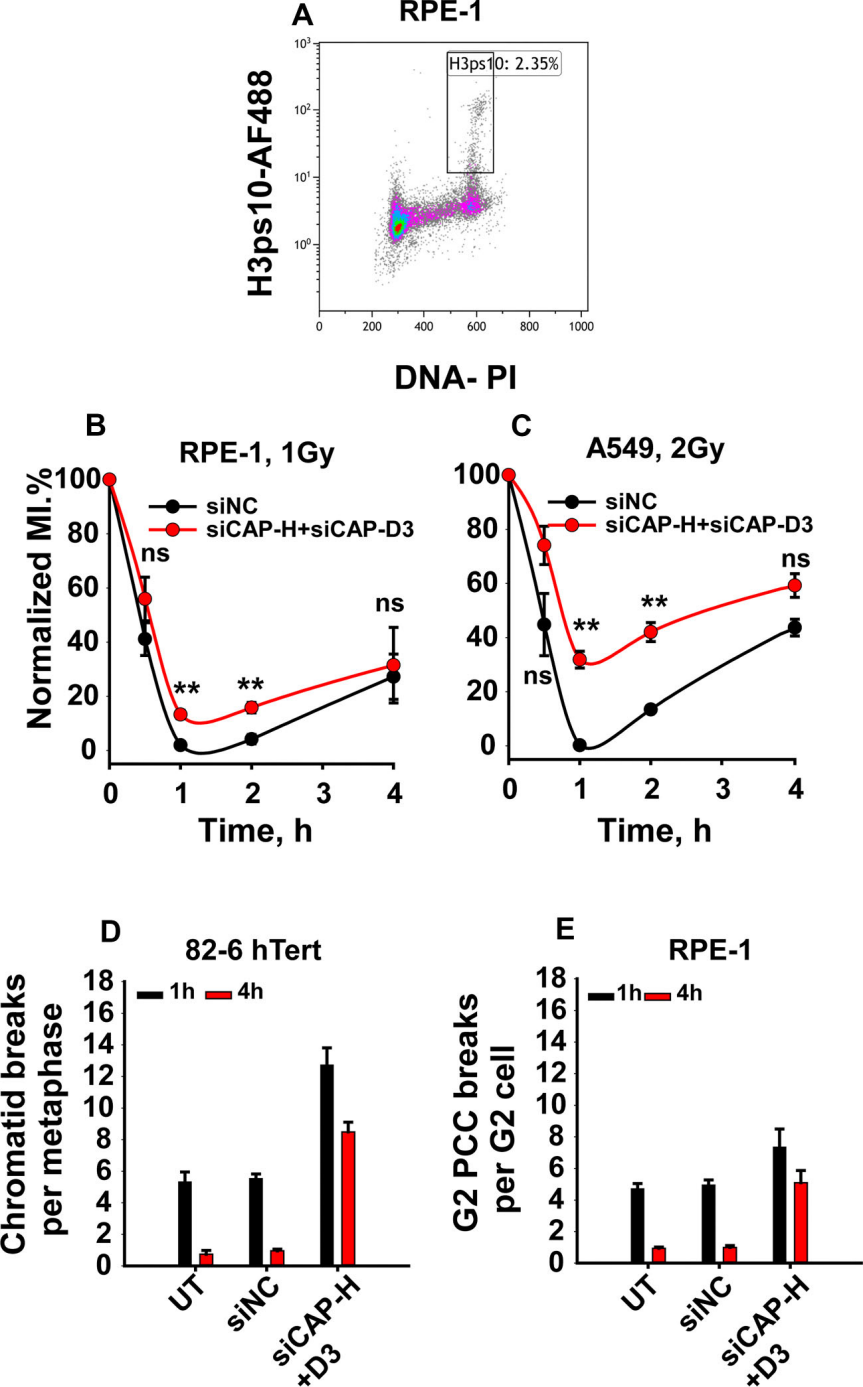
Impact of condensin depletion on G_2_-checkpoint and repair of metaphase and G_2_-PCC breaks **(A)** Representative two-parametric FACS histograms depicting the mitotic index (MI) to evaluate G_2_-checkpoint in RPE-1 cells; **(B)** Normalized MI in control and condensin-depleted RPE-1 cells. **(C)** As in panel **(B)**, but for A549 cells. **(D)** Kinetics of chromatid breaks at indicated times post-IR in control and condensins-depleted 82–6 hTert cells. **(E)** Kinetics of PCC breaks at indicated times post-IR in control and condensin-depleted RPE-1 cells. G_2_-checkpoint data represent the mean ± SD from two experiments, and cytogenetics data represent the mean ± SD from three independent experiments.

The G_2_ checkpoint delays mitotic entry to allow for DNA repair and genome stability [47]. Because HR is crucial for G_2_ checkpoint activation [42] and HR is impaired in condensins-depleted cells, we assessed checkpoint function via phospho-H3Ser10 (H3pS10) analysis using two-parameter flow cytometry (Fig. 7A). Control cells displayed robust checkpoint activation post-IR, with mitotic index (MI) recovery at 2–4 h (Fig. 7 B and C). Condensin knockdown caused only a minor checkpoint defect in RPE-1 cells (Fig. 7B) but a more pronounced defect in A549 cells (Fig. 7C), suggesting a stronger impact on tumor cells. Kinase inhibitors were used to examine whether ATM and ATR signaling, required for the checkpoint, were affected after condensin knockdown. Inhibition of either ATM or ATR alone or in combination fully abrogated the residual G_2_ checkpoint in condensins-deficient RPE-1 cells (Supplementary Figs. S11C–E), suggesting that upstream checkpoint signaling remains intact.

### Condensin suppression impairs chromosome break repair in the G2 phase of the cell cycle

Chromosomal DSBs that escape repair manifest as chromosome breaks and represent a major cause of radiation-induced cell death [48]. Previous studies showed that HR is essential for chromosome break repair for cells irradiated in G_2_-phase [39, 48]. To assess chromosome damage, we analyzed chromatid breaks at metaphase, as well as in G2-phase after calyculin A-induced PCC [49].

In metaphase preparations of 82–6 hTert cells irradiated in G_2_-phase (Supplementary Figs S12A–B), condensin depletion caused visible chromosome decondensation (Supplementary Figs  S12C and D), but chromosome break scoring was still possible. Compared to control cells, condensin-depleted cells exhibited ∼2-fold more breaks and significantly reduced repair efficiency (∼30% versus >80%; Fig. 7D), in line with the observed suppression of HR. These findings underscore the essential role of condensins in metaphase chromosome break repair.

To carry out an analysis of chromosome break repair even during the time of arrest in G_2_-phase, we employed the PCC assay (Supplementary Fig. S12E–H). PCC break repair was efficient in control RPE-1 cells, with more than 80% of the breaks detected at 1 h being repaired after 4 h. Strikingly, after condensin knockdown, only about 30% of the PCC breaks were repaired after the same incubation time (Fig. 7E). Initial PCC breaks were also elevated (∼1.3-fold) after condensin knockdown.

## Discussion

DNA in eukaryotes is tightly packaged to form chromatin, which presents a physical barrier to DSB repair-protein recruitment; therefore, chromatin remodeling is a prerequisite for successful DNA repair. It has been reported that a controlled localized chromatin unfolding occurs at DSBs to facilitate repair, while the bulk genome is rapidly compacted to prevent further damage to chromatin [50]. SMC complexes, cohesins, and condensins are the key elements of higher-order chromatin compaction [51].

Our results suggest that disruption of condensins poses a significant challenge to the DSB repair machinery by inhibiting DNA end resection and consequently suppressing HR, SSA, and alt-EJ. The effect of condensins is likely linked to the associated uncontrolled chromatin opening in the bulk genome. This implies that chromatin decondensation occurs in a tightly regulated manner where condensins may play a critical role. We demonstrate that they facilitate repair by resection-dependent pathways, particularly error-free HR. Notably, condensin I and II compensate for each other, and simultaneous knockdown of both condensins is required to achieve a measurable effect on radiosensitization, DSB repair, and DDR signaling. Our findings, together with cytogenetic evidence of marked chromosome decondensation in G2/M upon condensin loss, reinforce the role of condensins in sustaining higher-order chromatin architecture [1, 3]. It is our working hypothesis that this structural change in chromatin likely underlies the altered dynamics of DSB repair in our study.

Condensin II has previously been implicated in HR without providing cell cycle engagement details [17, 52]. We uncovered here that both condensin I and II play crucial and possibly redundant roles in HR, specifically for cells irradiated in the G_2_-phase of the cell cycle. Strikingly, repair in cells irradiated in S-phase remains unaffected after condensin knockdown. The molecular details of this intriguing cell cycle effect remain to be elucidated, but it is likely to derive from the altered cross-talk between ATM and ATR [43]. Additionally, condensin knockdown inhibited DSB end resection in G_2_-phase and also other resection-dependent pathways such as SSA, and alt-EJ

The observed G_2_-phase specificity aligns with the hypothesis that condensins play a critical role in coordinating chromatin architecture with the DNA damage response post replication, and may be linked to the role of condensins in mitosis. In G_2_-phase cells, where chromatin undergoes preparatory restructuring for mitosis, condensins may facilitate repair by promoting efficient chromatin compaction or aiding the recruitment and retention of repair factors at DSB sites. Vice versa, condensin suppression may disrupt the spatial organization required for HR, the predominant repair pathway in G_2_.

We have previously reported similarly striking differences between replicated (G_2_-phase) and replicating (S-phase) chromatin in other DDR responses. For instance, ATM and ATR jointly regulate resection and the G_2_ checkpoint in replicated G_2_ chromatin [43], acting cooperatively and epistatically at low IR doses. In contrast, during S-phase, they operate independently and pursue distinct functional goals [53]. Likewise, SKP2 depletion markedly suppresses resection and HR in G_2_, much like condensin loss, yet it has no detectable effect when cells are irradiated during DNA replication [54]. At present, the mechanistic basis of these cell-cycle-specific differences remains unclear, but we are developing models and experimental strategies to uncover their origins, while focusing on changes in the functional organization of the DNA-PKcs/ATM/ATR module [43, 53, 55].

The G_2_-specific phenotype observed here is unlikely to arise from differences in expression or intrinsic activity of the core resection machinery (Fig. 6), suggesting that condensins influence DSB processing through mechanisms independent of the nucleases themselves. One possibility is that G_2_-phase chromatin organization, or higher-order topology, creates a structural context in which condensins function and becomes uniquely important for regulating end resection. Additionally, G_2_-specific post-translational modifications of condensins or associated chromatin factors may modulate their ability to facilitate or restrict access of resection enzymes to DNA ends. Finally, G_2_-enriched regulatory pathways, such as cell cycle–controlled chromatin remodelers or checkpoint kinases, may further shape condensin-dependent control of DNA end processing. Future studies will determine which of these mechanisms underlie the observed G_2_ specificity.

Both condensins and cohesins belong to a similar class of proteins (SMC) that shape the higher-order chromatin structure throughout the cell cycle [51]. The strong suppression of resection and HR by condensin depletion suggests that, without functional condensins, cohesins alone may not be sufficient to maintain optimal chromatin state for DSB repair. The phenotype observed upon condensin depletion, delayed DSB repair, and increased radiosensitivity, closely resembles that reported for cohesin loss in G_2_ phase [56, 57]. Given cohesin’s established role in facilitating DSB repair and its regulated recruitment to damage sites [58, 59], these parallels suggest a functional interplay between cohesins and condensins in the G_2_-phase DNA damage response and merit further investigation.

Classically, condensins have been implicated in chromatin organization during mitosis [51]. Recently, however, condensins have also been shown to be important in chromatin organization during interphase [11]. Our data suggest that condensins indeed play an important role in chromatin organization during interphase, particularly in G_2_ phase cells, where condensins suppression upon irradiation compromises resection and DSB repair. The fuzzy appearance of metaphase chromosomes upon condensin knockdown (Supplementary Fig. S3G, S12C and D) is indicative of their classical role in maintaining metaphase chromosome compaction. However, it is noteworthy that chromosome breaks were still microscopically visible in these sub-optimally condensed metaphase chromosomes (Supplementary Fig. S12D).

Interestingly, condensin knockdown disturbs the balance among DSB repair pathways by specifically suppressing resection-dependent repair while leaving c-NHEJ largely unaffected. These results highlight the complex and multifaceted roles of condensins in DNA repair. Testing the radiosensitizing effect of combined suppression of condensins and c-NHEJ may be a useful approach to further enhance radiosensitization in tumor cells, and thus widen the therapeutic window in the clinic.

Our results also raise intriguing questions as to the proper function of condensins for cell survival. The marked impairment of DSB repair in G_2_-phase cells suggests that condensin suppression sensitizes cells to IR in a cell cycle-specific manner. Future studies should assess cell survival in G_1_/G_0_ plateau-phase and in synchronized G_2_-phase cells to validate this hypothesis and elucidate the broader implications of condensin function in cell radiosensitivity.

We have previously reported a direct correlation between functional HR and IR-induced G_2_ checkpoint and G_2_ chromosome break repair at low doses of IR [42, 48]. Cell cycle checkpoints are frequently dysregulated in cancer cells [60], and chromosome breaks directly feed into IR-induced cell killing. Strikingly, we observed a strong suppression of G_2_ chromosome break repair and a noticeable abrogation of the G_2_ checkpoint upon condensins knockdown. These results validate our previous findings that HR suppression strongly inhibits IR-induced G_2_ chromosome break repair and the G_2_ checkpoint [42, 48]. The observed defect in chromosome break processing in condensin-depleted cells underscores the importance of condensins for efficient error-free repair by maintaining optimal chromosome compaction, ensuring proper alignment and accessibility of DSB repair machinery at the damage site.

Recent studies have linked specific condensin subunits to oncogenic signalling and therapy resistance, underscoring their emerging relevance in cancer biology. NCAPD3 activates the AKT pathway in prostate cancer [61], while NCAPH modulates breast cancer progression and treatment response [62]; it has also been implicated in chromosome condensation and DNA damage response in pancreatic cancer cells [63]. Our data further demonstrate that condensin depletion in G_2_-phase cells impairs DSB repair and increases radiosensitivity - all hallmarks of HR defects [64]. This phenotypic overlap suggests that condensin dysfunction may simultaneously disrupt DNA repair and modulate oncogenic pathways, thereby contributing to cancer development and therapeutic resistance.

Despite efforts to develop small-molecule inhibitors against condensin subunits in E. coli [65], no vertebrate-specific inhibitors have been established yet. Given the dysregulated expression of condensin I and II in various human cancers [66–68], targeting these complexes may offer novel therapeutic avenues. The G_2_-specific defects observed here highlight the potential of combining condensin inhibition with RT, which is anticipated to spare normal tissues, most of which are non-cycling and thus less susceptible to condensin-targeted interventions. Collectively, these findings emphasize condensin’s dual role in maintaining genome integrity and regulating cancer signaling, while pointing toward its therapeutic potential in precision oncology.

While our study provides important insights into the role of condensins in shaping the DSB repair pathway choice, it does not directly examine how the loss influences chromatin accessibility, resection dynamics, or the recruitment of specific repair factors to DSBs. These mechanistic aspects remain open questions and represent key directions for future work, which will build on the framework established here. By acknowledging these gaps, we highlight the need for deeper molecular dissection of condensin-dependent regulation of DSB repair pathways.

## Conclusion

Condensins play a crucial role in maintaining chromatin architecture during mitosis, as well as in interphase cells. We report here for the first time the importance of condensins in IR-induced DSB repair, specifically in the G_2_ phase of the cell cycle. We uncover that condensins help to maintain genomic stability by supporting HR and other resection-dependent pathways. The mechanistic basis behind these potent effects warrants further investigation.

## Supplementary Material

gkag076_Supplemental_File

## Data Availability

The data underlying this article are available in the article and its online supplementary material.
