# Correction to ‘Condensins regulate resection–dependent DNA double–strand break repair pathways in replicated chromatin’

**DOI:** 10.1093/nar/gkag164

**Published:** 2026-02-23

This is a correction to: Mei Liu, Wei You, Lisa-Marie Weber, Emil Mladenov, Xixi Lin, Veronika Mladenova, Ramtin Omid Shafaat, Gabriel E Pantelias, Eleni Gkika, Martin Stuschke, Aashish Soni, George Iliakis, Condensins regulate resection–dependent DNA double–strand break repair pathways in replicated chromatin, *Nucleic Acids Research*, Volume 54, Issue 4, 27 February 2026, gkag076, https://doi.org/10.1093/nar/gkag076

The second author’s names have been emended to read: “Wei You”.

