# Supplementary material for: Condensins regulate resection–dependent DNA double–strand break repair pathways in replicated chromatin
Source: Nucleic Acids Res. 2026 Feb 9;54(4):gkag076. doi: 10.1093/nar/gkag076 (PMC12884081; doi:10.1093/nar/gkag076)
Supplement: gkag076_Supplemental_File [file gkag076_supplemental_file.pdf]

## Supplementary Figure Legends

**Figure S1**. *Knockdown efficiency test* and *the impact of condensins depletion on cell cycle distribution.* (A) The depletion level of CAP-H in RPE-1 cells was determined at 48 h after transfection using four different siRNAs and their combination. (B) The depletion levels of SMC2 in RPE-1 cells were determined at 48 h after transfection using four different siRNAs. (C) Cell cycle distribution of RPE-1 cells upon single depletion of CAP-H or CAP-D3. (D) Distribution of RPE-1 cells in different cell cycle phases upon single depletion of CAP-H or CAP-D3. (E) Same as panel (C), but for 82-6 hTert cells. (F) Same as panel (D), but for 82-6 hTert cells. Data in (D) and (F) represent the mean ± SD from three independent experiments

**Figure S2.** *Impact of Condensins depletion on cell cycle distribution***.** (A) Cell cycle distribution of RPE-1 cells upon combined depletion of CAP-H and CAP-D3. (B) Distribution of RPE-1 cells in different cell cycle phases upon combined depletion of CAP-H and CAP-D3. (C) Same as Panel (A), but for 82- 6 hTert cells. (D) Same as panel (B), but for 82-6 hTert cells. (E) Same as panel (A), but for A549 cells. (F) Same as panel (B), but for A549 cells. Data represent the mean ± SD from three independent experiments.

**Figure S3.** *SMC2 knockdown severely compromises plating efficiency in both normal and tumor cell lines.* (A) Western blot analysis of SMC2 depletion in RPE-1 cells at 48 h post-transfection. (B) Same as panel (A), but for 82-6 hTert cells. (C) Same as panel (A), but for A549 cells. (D) Representative images of clonogenic survival of RPE-1 cells upon SMC2 depletion. (E) As panel (D), but for 82-6 hTert cells. (F) As panel (D), but for A549 cells. (G) Representative M-FISH images in control and SMC2 knockdown RPE-1 cells.

**Figure S4.** *Impact of SMC2 depletion on cell cycle distribution*. (A) Cell cycle distribution of RPE-1 cells upon SMC2 depletion. (B) Impact of SMC2 knockdown on the distribution of RPE-1 cells in different cell cycle phases. (C-D) Same as panel A-B, respectively, but for 82-6 cells. (E-F) Same as panel A-B, respectively, but for A549 cells. Data for RPE-1 cells represent the mean ± SD from three independent experiments, and for 82-6 hTert and A549, a single experiment.

**Figure S5**. *Impact of condensins depletion on the kinetics of γH2AX and 53BP1 foci in asynchronous A549 cells.* (A) Representative images of γH2AX foci in cells, irradiated with 1 Gy X-rays and collected at 1 h post-IR; (B) Kinetics of γH2AX foci in asynchronous cells depleted of condensins. (C) Representative images of 53BP1 foci in cells, irradiated with 1 Gy X-rays and collected at 1 h post-IR. (D) Kinetics of 53BP1 foci in A549 asynchronous cells depleted of condensins. Data represent the mean ± SD from three independent experiments.

**Figure S6**. *Impact of condensins depletion on the kinetics of γH2AX and 53BP1 foci in $G_1$, S, and $G_2$ phase A549 cells.* (A) Representative histograms obtained by QIBC (left panel) and the exemplary gating applied to QIBC dot plots for the evaluation of γ-H2AX, 53BP1, RPA70, or RAD51 foci in a cell-cycle-specific manner (right panel) in RPE-1 cells. (B) Kinetics of γH2AX foci in $EdU^-$, $G_1$-phase, A549 cells depleted of condensins. (C) Kinetics of γH2AX foci in $EdU^+$, S-phase, A549 cells depleted of condensins. (D) $G_2$-specific quantification of γ-H2AX foci in A549 cells upon depletion of condensins. (E) Kinetics of 53BP1 foci in $EdU^-$, $G_1$-phase, A549 cells depleted of condensins. (F) Kinetics of 53BP1 foci in $EdU^+$, S-phase, A549 cells depleted of condensins. (G) $G_2$-specific quantification of 53BP1 foci in A549 cells upon depletion of condensins. Data represent the mean ± SD from three independent experiments.

**Figure S7**. *Impact of condensin depletion on the kinetics of RAD51 foci in A549 cells.* (A) Representative images of Rad51 foci in A549 cells, irradiated with 2 Gy of X-rays and collected at 3h post irradiation; (B) Kinetics of Rad51 foci in $EdU^-$, $G_1$-phase cells. (C) Kinetics of Rad51 foci in $EdU^+$, S-phase cells. (D) $G_2$-specific quantification of Rad51 foci upon Condensins depletion; Data represent the mean ± SD from three independent experiments.

**Figure S8**. *Impact of condensins depletion on the kinetics of RPA70 foci in A549 cells.* (A) Representative images of RPA70 foci in RPE-1 cells, irradiated with 2 Gy of X-rays and collected at 3h post irradiation; (B) Kinetics of RPA70 foci in $EdU^-$, $G_1$-phase cells. (C) Kinetics of RPA70 foci in $EdU^+$, S-phase cells. (D) $G_2$-specific quantification of RPA70 foci upon condensins depletion; Data represent the mean ± SD from three independent experiments.

**Figure S9.** (A) Western blot analysis of CAP-H and CAP-D3 protein levels in DR-GFP cells at 48 h post-transfection of siCAP-H + siCAP-D3; GAPDH served as a loading control. (B) As in panel (A), but for SA-GFP cells. (C) As in panel (A), but for EJ2-GFP cells. (D) As in panel (A), but for EJ5-GFP cells. (E) Kinetics of DSB repair measured by PFGE in siNC or siCAP-H + siCAP-D3 transfected A549 cells irradiated with 10Gy X-rays in the presence or absence of DNA-PKcs inhibitor, NU7441. PFGE data represent mean ± SD from two independent determinations.

**Figure S10.** *Western blot and flow cytometry data for cell sorting in G1, S, and G2 phases in A549 cells*

(A) Knockdown efficiency of condensins in exponentially growing A549 cells, which were used for the sorting experiment. (B) FACS histograms showing sorted $G_1$, S, and $G_2$ enriched populations in cells transfected with control siRNA, as well as the gates applied for sorting. (C) As in panel (B), for cells depleted of condensins.

**Figure S11.** (A) Knockdown efficiency of CAP-H measured in sorted $G_1$, S, and $G_2$ populations of A549 cells measured by q-RT-PCR. (B) Same as in panel (A), but for the knockdown efficiency of CAP-D3. (C) Normalized MI of siNC-transfected RPE-1 cells after treatment with 10 µM KU55933 (ATMi) or 5 µM VE-821 (ATRi). (D) Normalized MI of condensin-depleted RPE-1 cells after treatment with ATRi. (E) Normalized MI of condensin-depleted RPE-1 cells after treatment with ATMi. $G_2$-checkpoint data represent the mean ± SD from two independent experiments, and cytogenetics

**Figure S12.** Chromatid breaks, and Calyculin A-induced $G_2$-PCC breaks were measured in exponentially growing Condensins-depleted 82-6hTert and RPE-1 cells upon exposure to 1 or 0.5 Gy X-rays, respectively. (A-B) Representative images of chromatid breaks (indicated with arrows) in the control cells. (C-D) Representative images of chromatid breaks (indicated with arrows) in Condensins-depleted cells. (E-F) Representative image of PCC breaks (indicated with arrows) in control cells. (G-H) Representative image of PCC breaks (indicated with arrows) in Condensins-depleted cells.

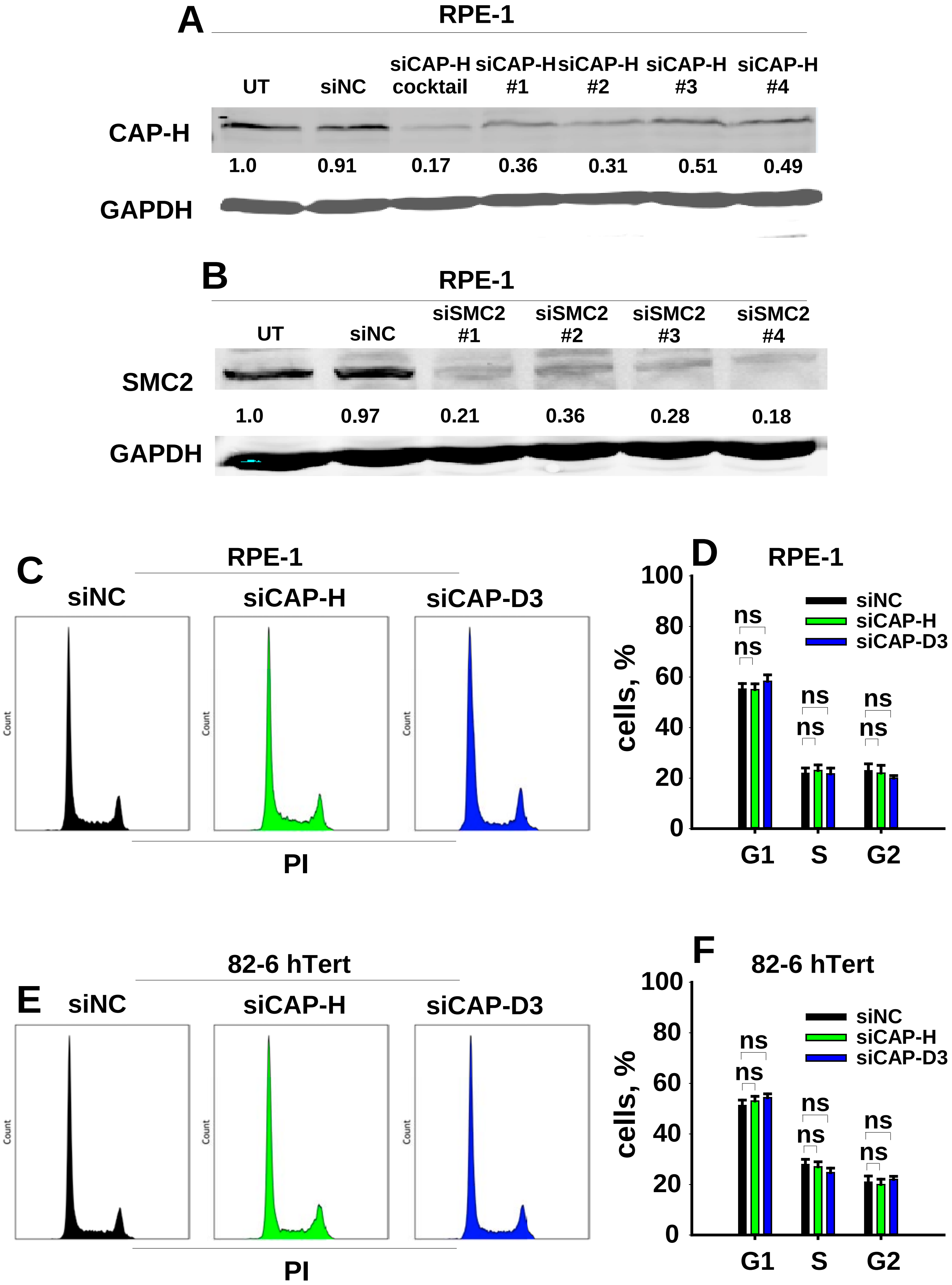
A
RPE-1
UT
siNC
siCAP-H cocktail
siCAP-H #1
siCAP-H #2
siCAP-H #3
siCAP-H #4
CAP-H
1.0
0.91
0.17
0.36
0.31
0.51
0.49
GAPDH
B
RPE-1
UT
siNC
siSMC2 #1
siSMC2 #2
siSMC2 #3
siSMC2 #4
SMC2
1.0
0.97
0.21
0.36
0.28
0.18
GAPDH
C
RPE-1
siNC
siCAP-H
siCAP-D3
PI
D
RPE-1
cells, %
100
80
60
40
20
0
ns
siNC
siCAP-H
siCAP-D3
G1
S
G2
E
82-6 hTert
siNC
siCAP-H
siCAP-D3
PI
F
82-6 hTert
cells, %
ns
siNC
siCAP-H
siCAP-D3
G1
S
G2

Figure S1

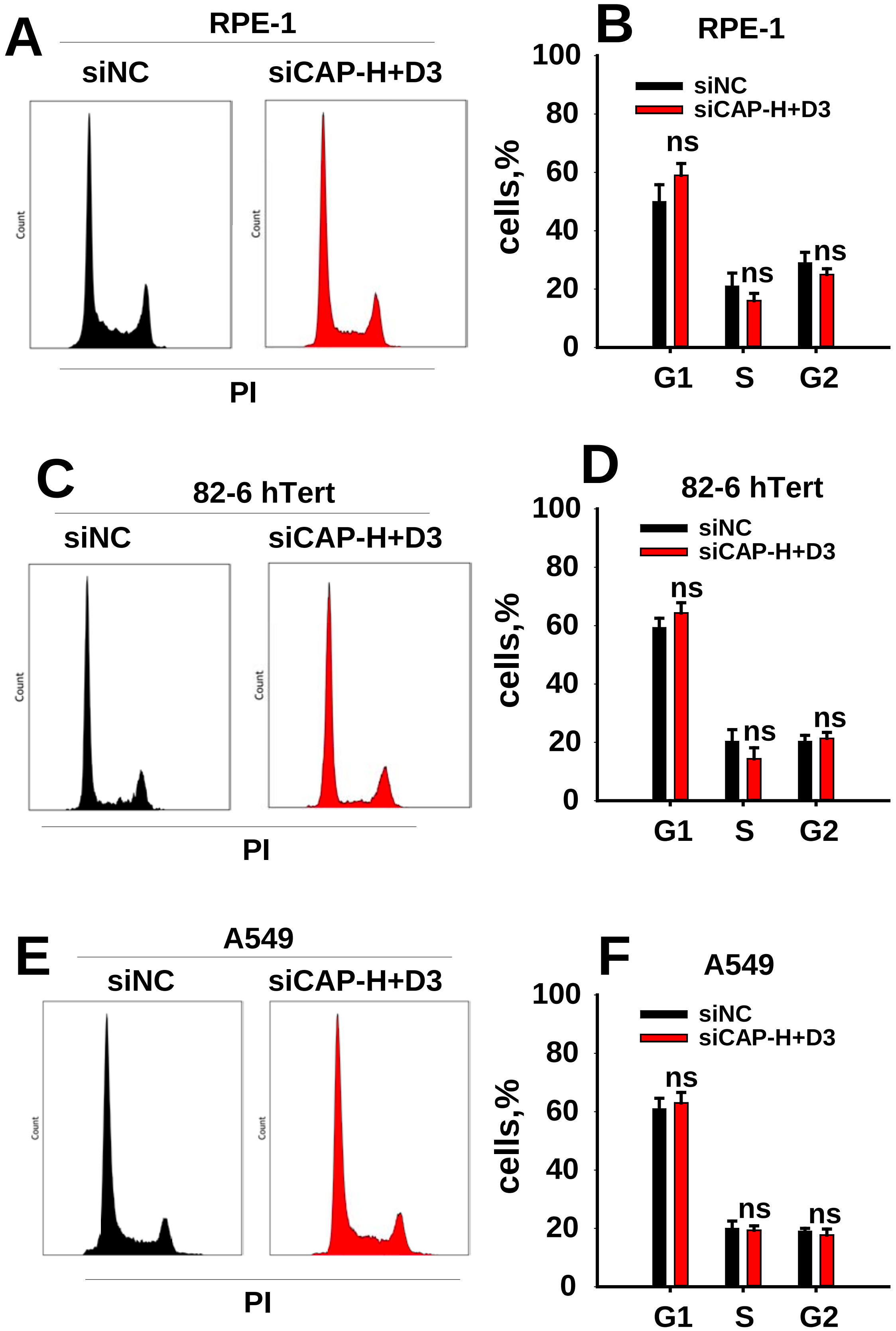
A
RPE-1
siNC
siCAP-H+D3
Count
PI
B
RPE-1
100
80
60
40
20
0
cells,%
siNC
siCAP-H+D3
ns
G1
S
G2
C
82-6 hTert
D
E
A549
F

# Figure S2

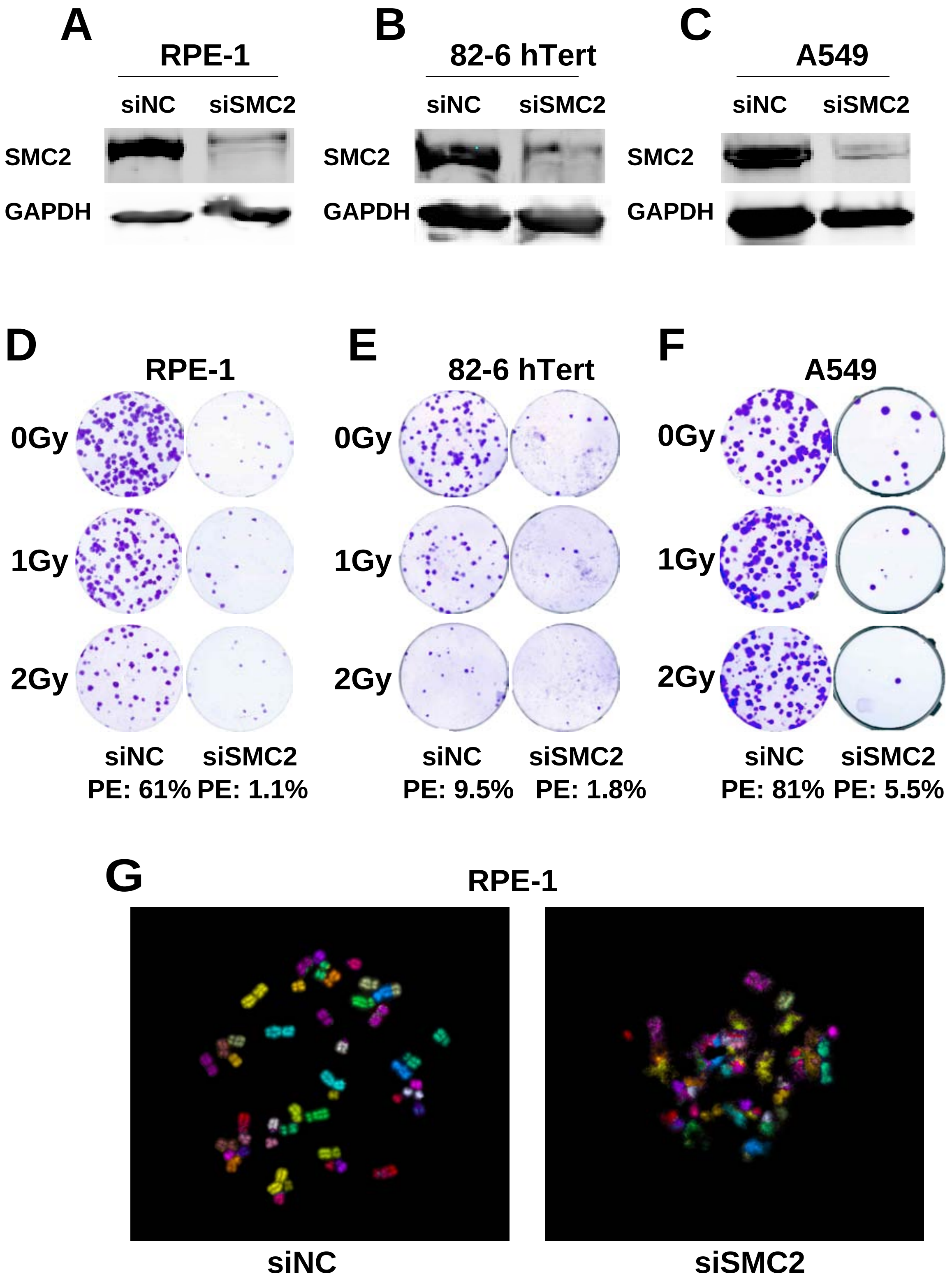
A
RPE-1
siNC siSMC2
SMC2
GAPDH
B
82-6 hTert
siNC siSMC2
SMC2
GAPDH
C
A549
siNC siSMC2
SMC2
GAPDH
D
RPE-1
0Gy
1Gy
2Gy
siNC siSMC2
PE: 61% PE: 1.1%
E
82-6 hTert
0Gy
1Gy
2Gy
siNC siSMC2
PE: 9.5% PE: 1.8%
F
A549
0Gy
1Gy
2Gy
siNC siSMC2
PE: 81% PE: 5.5%
G
RPE-1
siNC
siSMC2

**Figure S3**

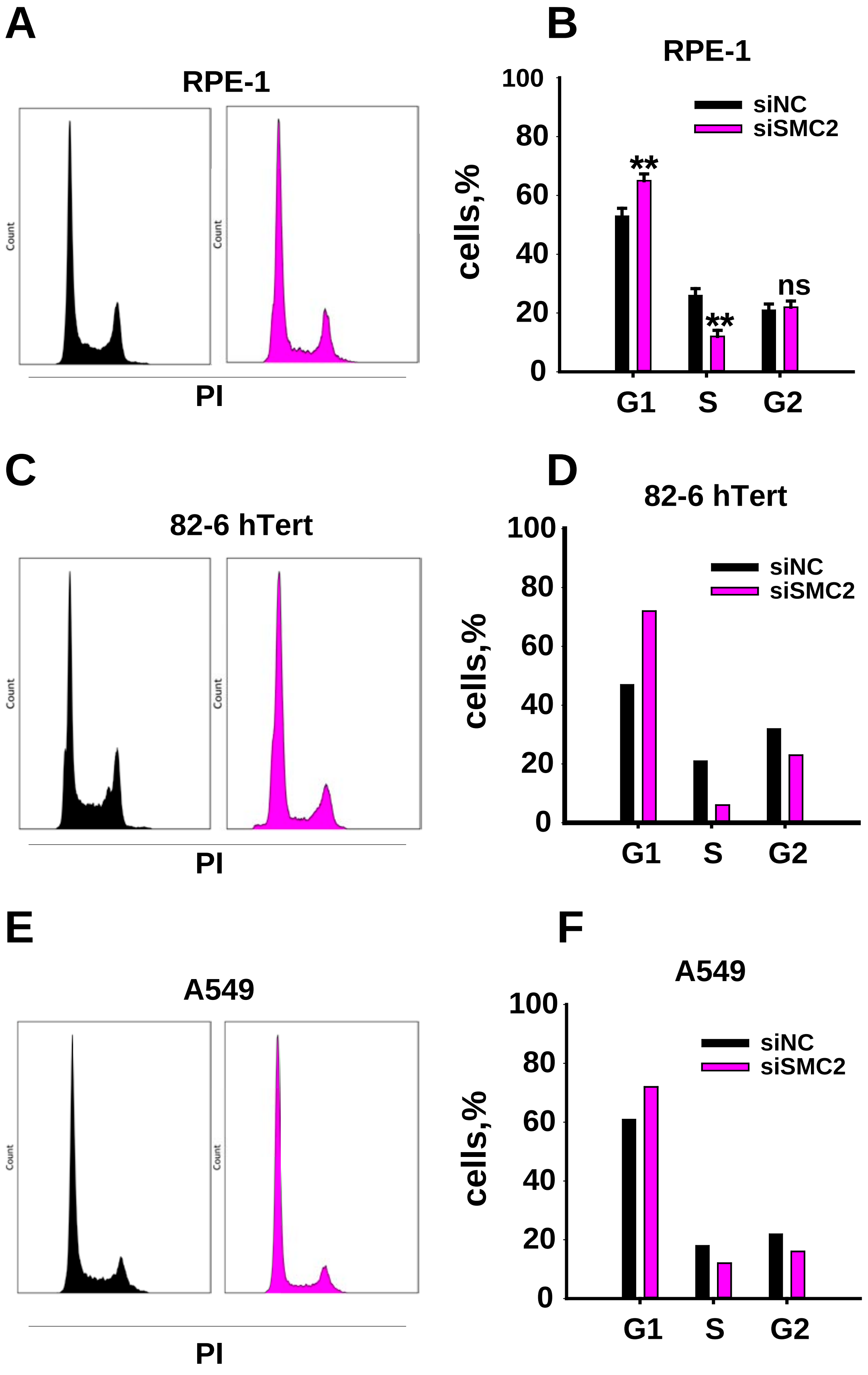
A
RPE-1
Count
Count
PI
B
RPE-1
100
80
60
40
20
0
cells,%
siNC
siSMC2
**
**
ns
G1
S
G2
C
82-6 hTert
Count
Count
PI
D
82-6 hTert
100
80
60
40
20
0
cells,%
siNC
siSMC2
G1
S
G2
E
A549
Count
Count
PI
F
A549
100
80
60
40
20
0
cells,%
siNC
siSMC2
G1
S
G2

# Figure S4

# A549

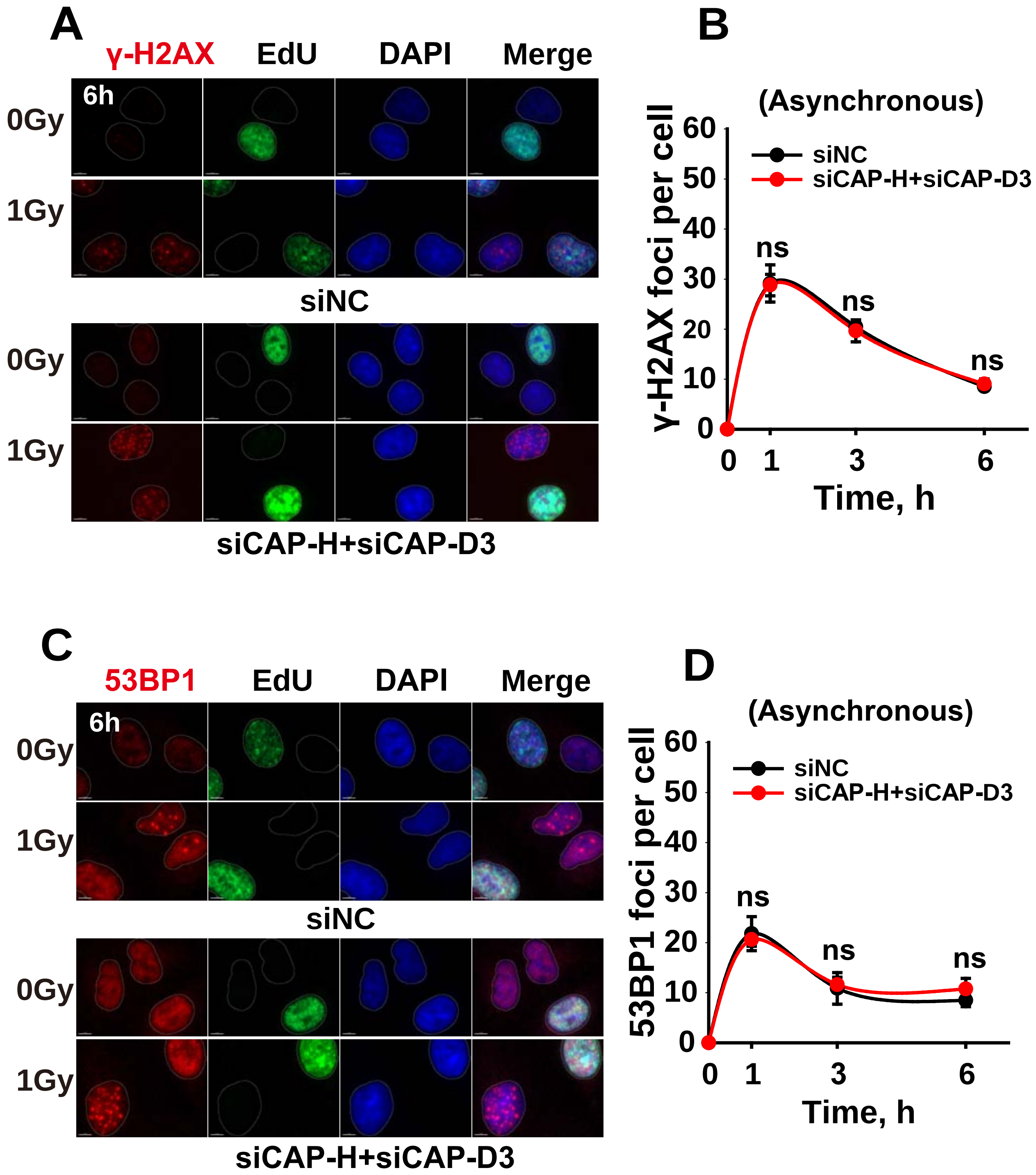


**Figure S5**

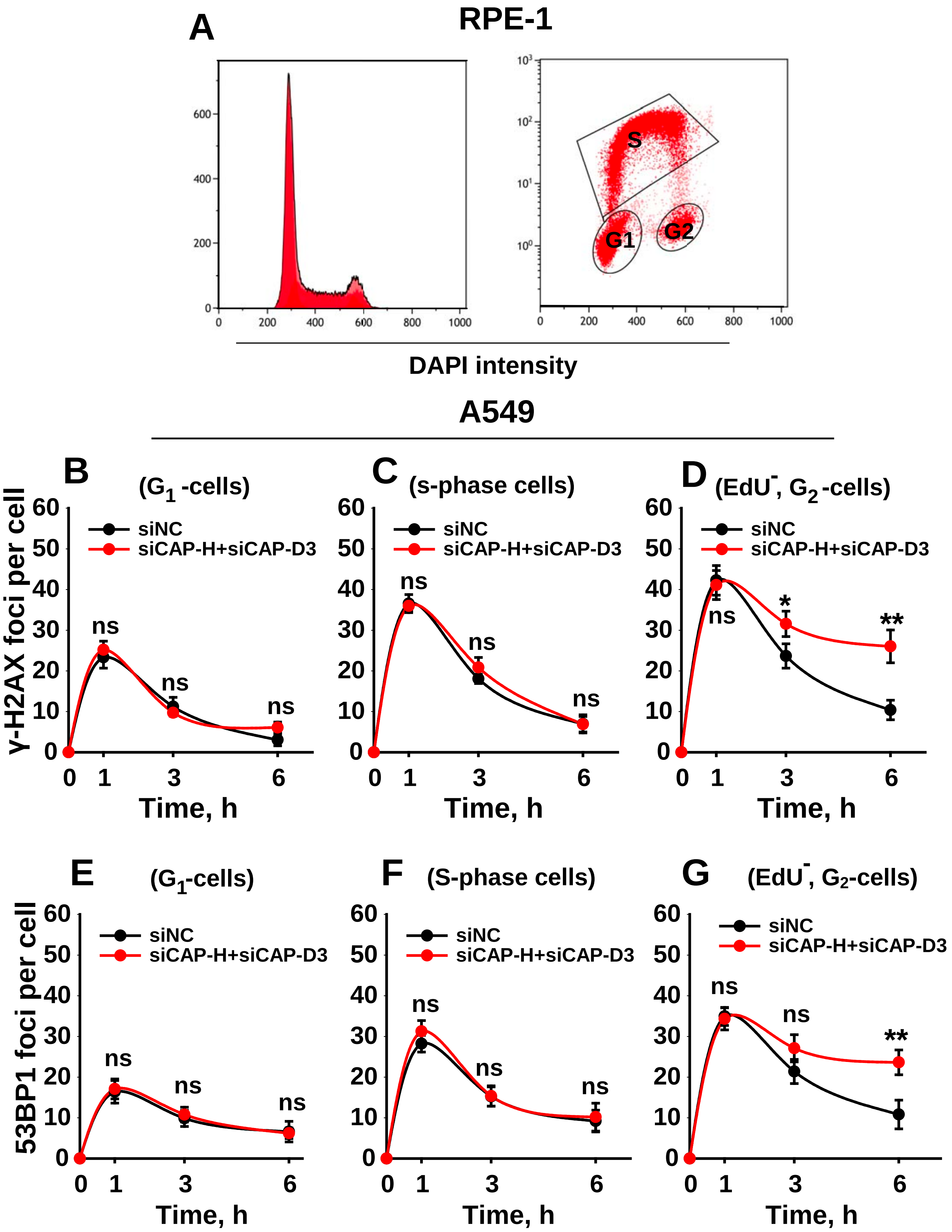

A
RPE-1
600
400
200
0
0
200
400
600
800
1000
10^3
10^2
10^1
10^0
S
G1
G2
DAPI intensity
A549
B
(G1 -cells)
C
(s-phase cells)
D
(EdU-, G2 -cells)
γ-H2AX foci per cell
siNC
siCAP-H+siCAP-D3
ns
*
**
60
50
40
30
20
10
0
1
3
6
Time, h
E
(G1-cells)
F
(S-phase cells)
G
(EdU-, G2-cells)
53BP1 foci per cell


Figure S6

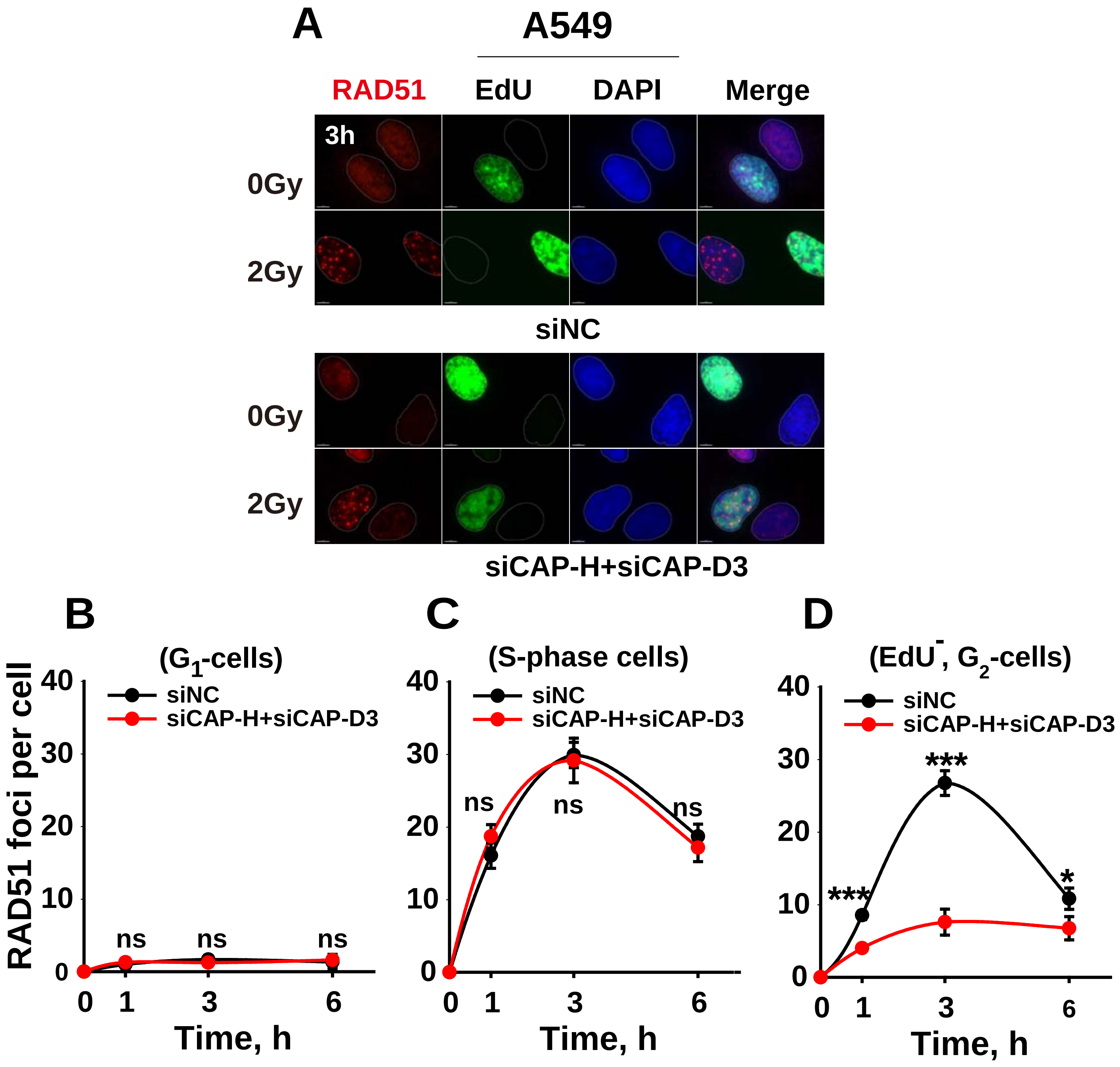
A
A549
RAD51
EdU
DAPI
Merge
3h
0Gy
2Gy
siNC
0Gy
2Gy
siCAP-H+siCAP-D3
B
(G1-cells)
siNC
siCAP-H+siCAP-D3
RAD51 foci per cell
ns
ns
ns
Time, h
C
(S-phase cells)
siNC
siCAP-H+siCAP-D3
ns
ns
ns
Time, h
D
(EdU-, G2-cells)
siNC
siCAP-H+siCAP-D3
***
***
*
Time, h

Figure S7

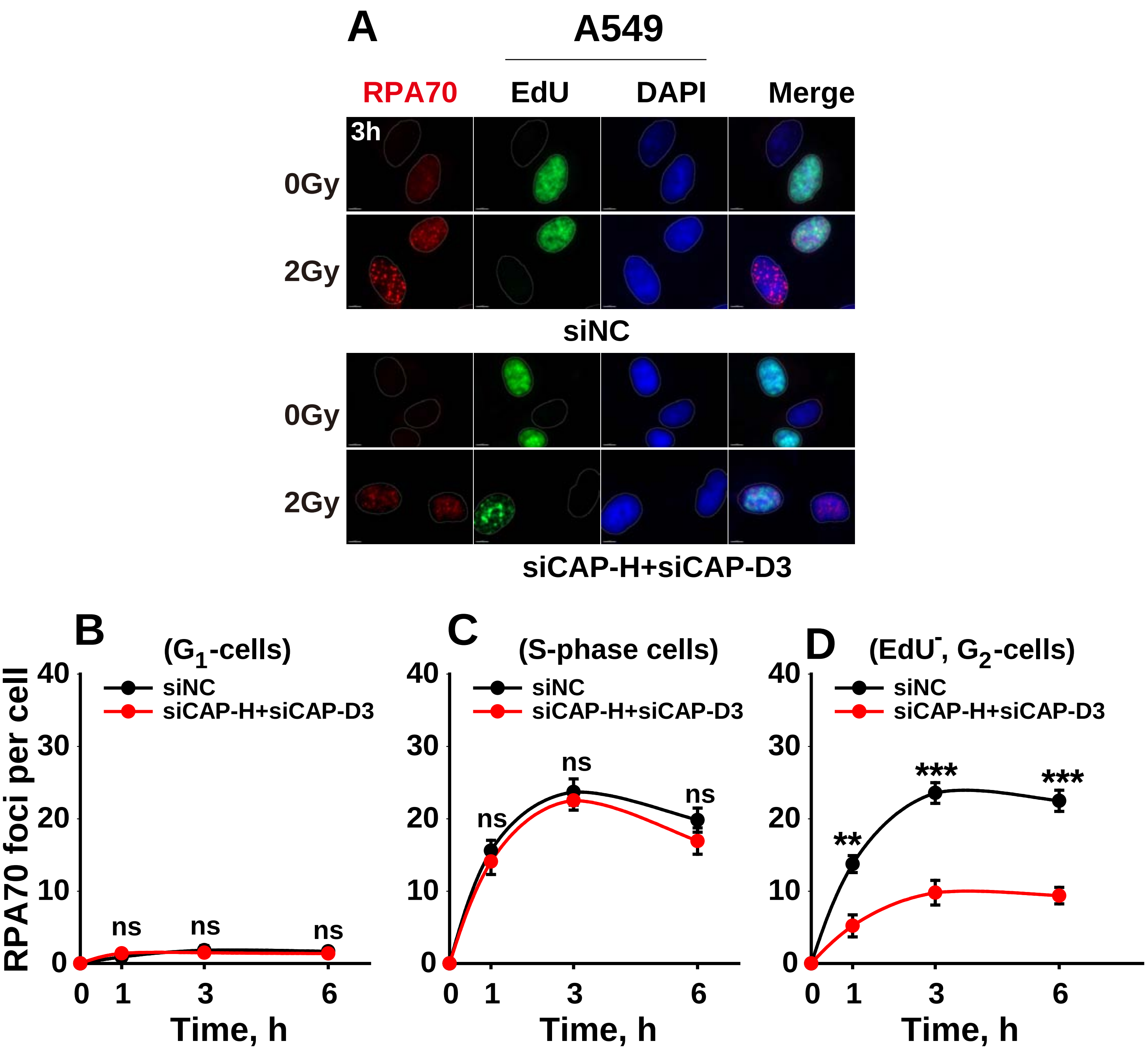

A
A549
RPA70
EdU
DAPI
Merge
3h
0Gy
2Gy
siNC
0Gy
2Gy
siCAP-H+siCAP-D3
B
(G1-cells)
RPA70 foci per cell
siNC
siCAP-H+siCAP-D3
ns
ns
ns
Time, h
C
(S-phase cells)
siNC
siCAP-H+siCAP-D3
ns
ns
ns
Time, h
D
(EdU-, G2-cells)
siNC
siCAP-H+siCAP-D3
**
***
***
Time, h


# Figure S8

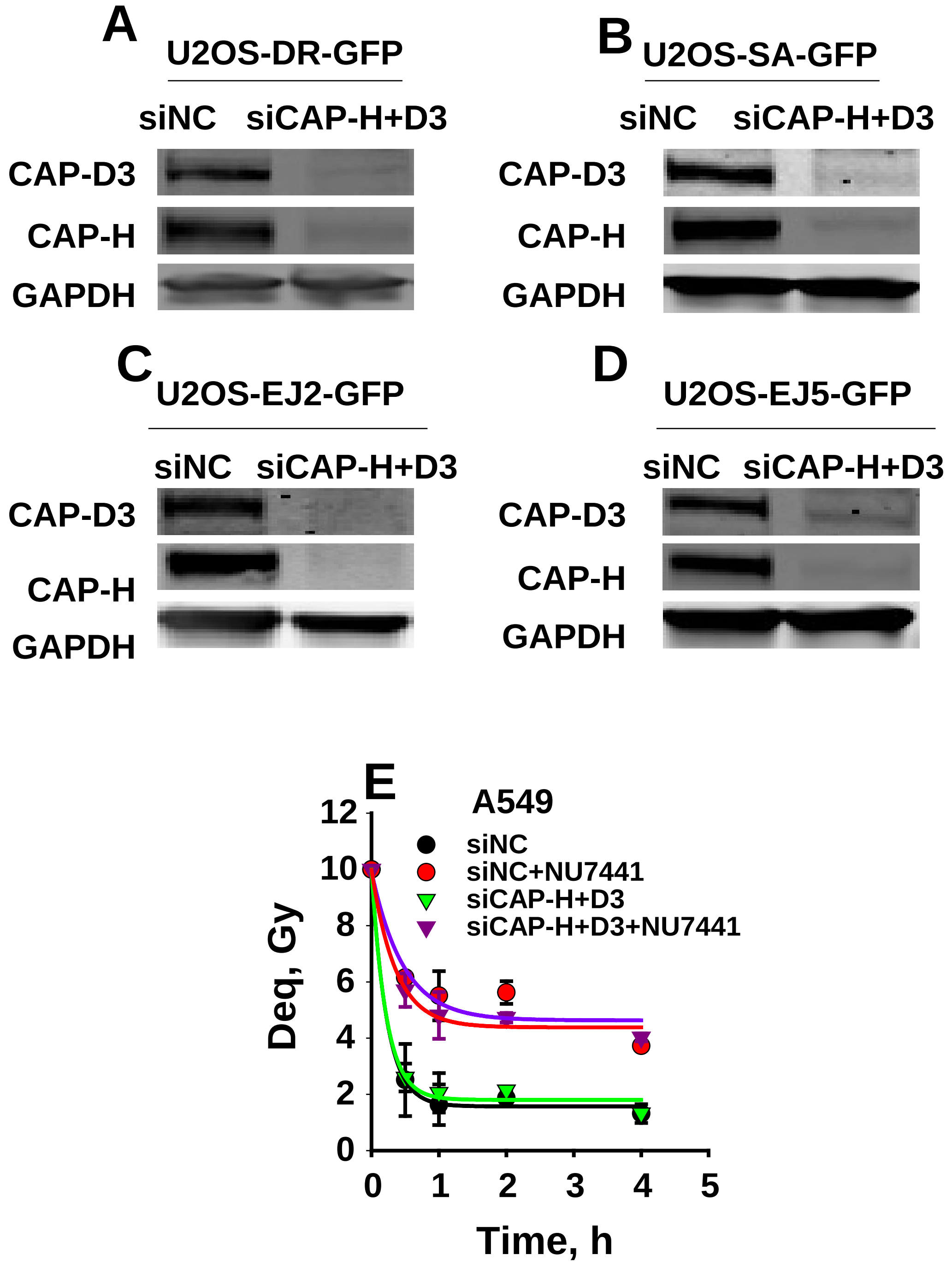
A
U2OS-DR-GFP
siNC
siCAP-H+D3
CAP-D3
CAP-H
GAPDH
B
U2OS-SA-GFP
siNC
siCAP-H+D3
CAP-D3
CAP-H
GAPDH
C
U2OS-EJ2-GFP
siNC
siCAP-H+D3
CAP-D3
CAP-H
GAPDH
D
U2OS-EJ5-GFP
siNC
siCAP-H+D3
CAP-D3
CAP-H
GAPDH
E
A549
siNC
siNC+NU7441
siCAP-H+D3
siCAP-H+D3+NU7441
Deq, Gy
Time, h
0
2
4
6
8
10
12
0
1
2
3
4
5

# Figure S9

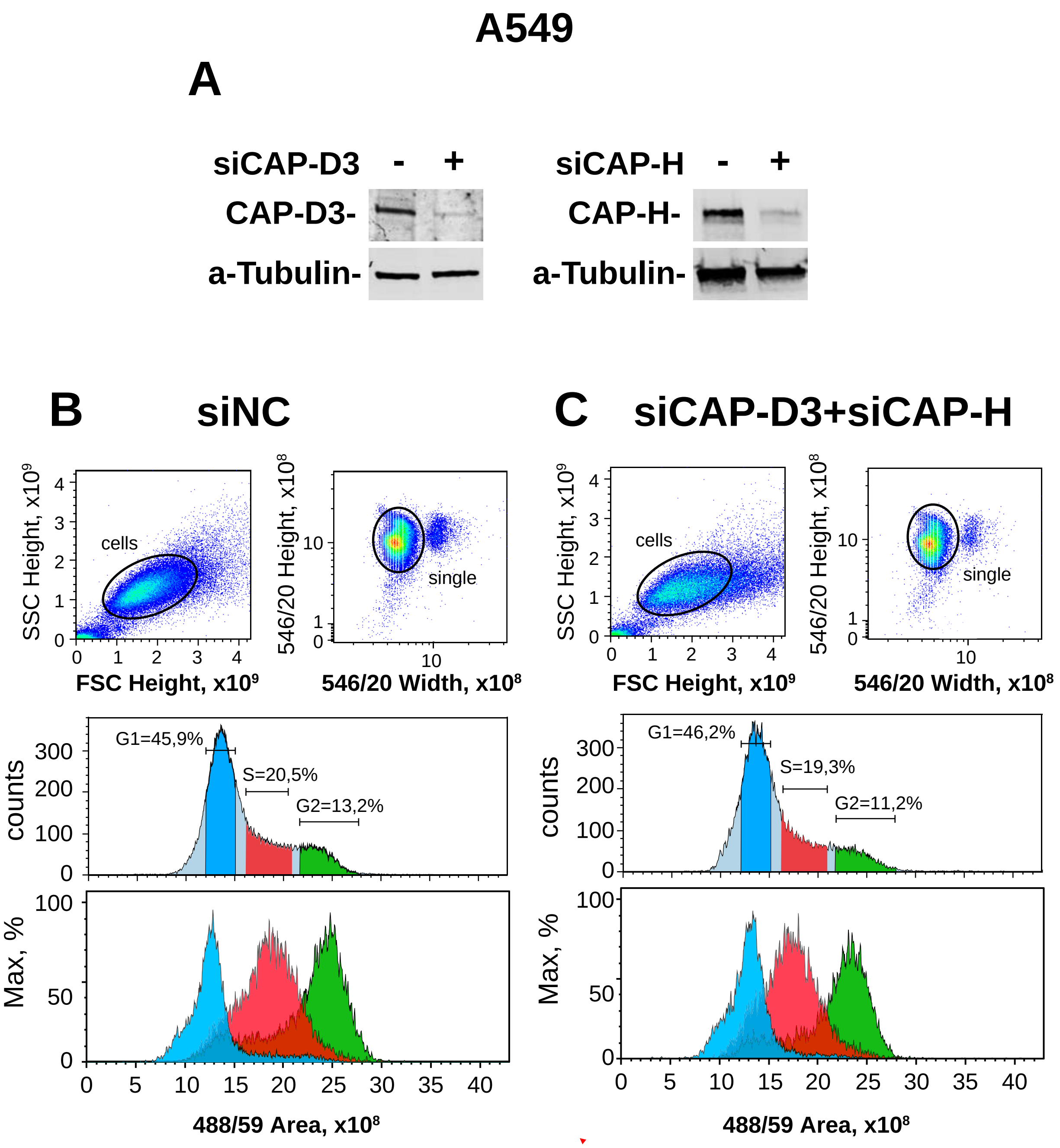
A549
A
siCAP-D3 - +
CAP-D3-
a-Tubulin-
siCAP-H - +
CAP-H-
a-Tubulin-
B
siNC
C
siCAP-D3+siCAP-H
SSC Height, x10⁹
FSC Height, x10⁹
cells
546/20 Height, x10⁸
546/20 Width, x10⁸
single
counts
G1=45,9%
S=20,5%
G2=13,2%
G1=46,2%
S=19,3%
G2=11,2%
Max, %
488/59 Area, x10⁸

# Figure S10

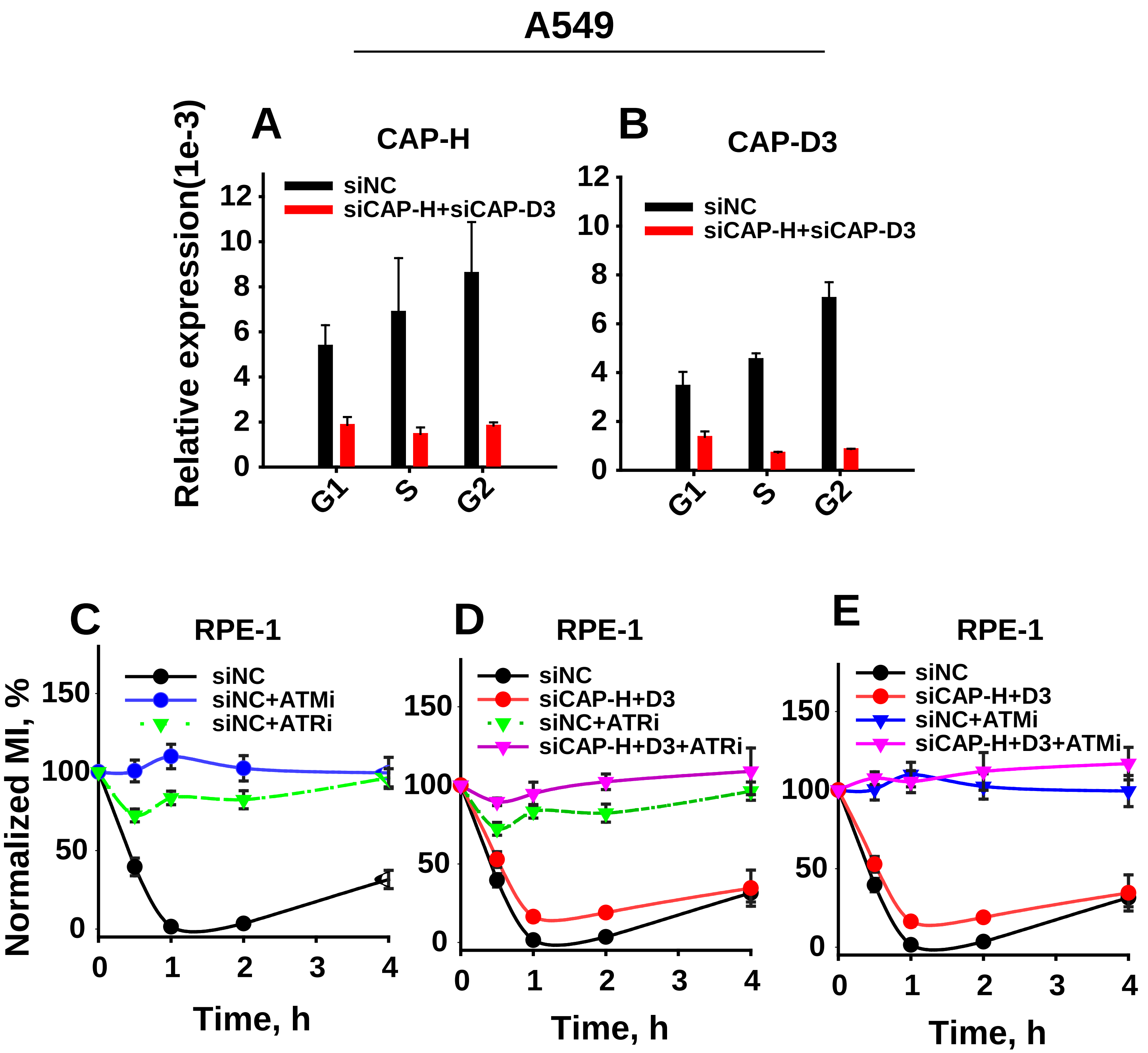
A549
A
CAP-H
Relative expression(1e-3)
siNC
siCAP-H+siCAP-D3
0
2
4
6
8
10
12
G1
S
G2
B
CAP-D3
siNC
siCAP-H+siCAP-D3
C
RPE-1
Normalized MI, %
siNC
siNC+ATMi
siNC+ATRi
0
50
100
150
Time, h
0
1
2
3
4
D
RPE-1
siNC
siCAP-H+D3
siNC+ATRi
siCAP-H+D3+ATRi
E
RPE-1
siNC
siCAP-H+D3
siNC+ATMi
siCAP-H+D3+ATMi

# Figure S11

## 82-6 hTert cells

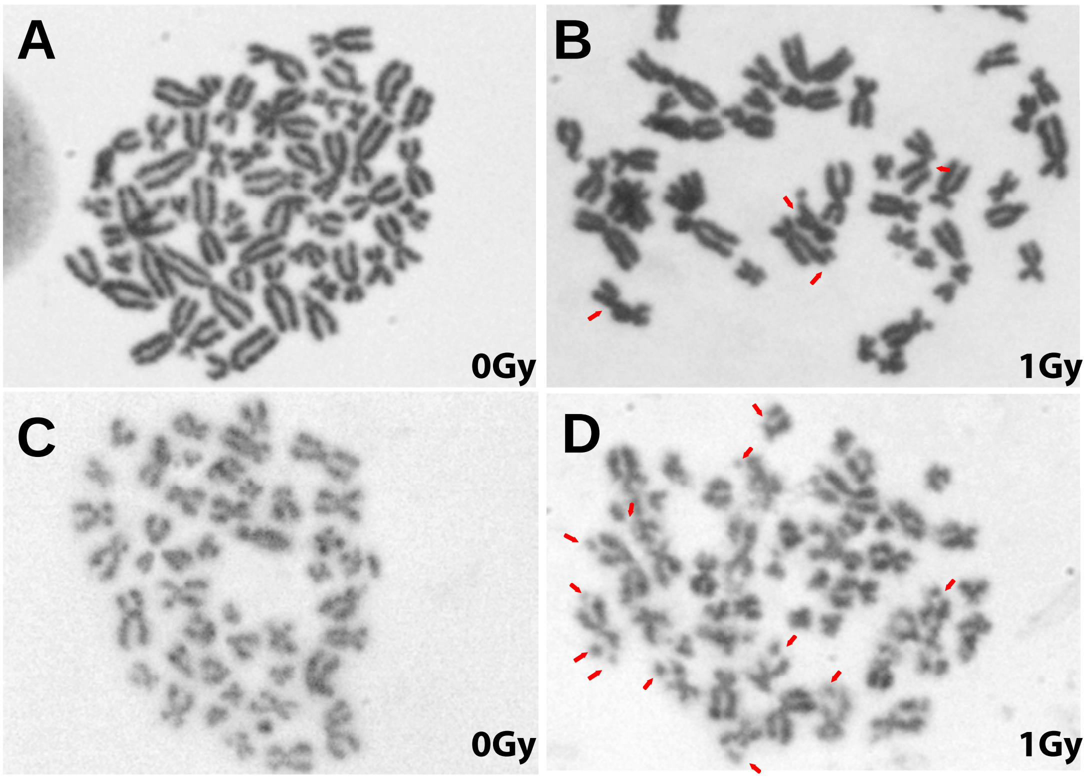


## RPE-1 cells

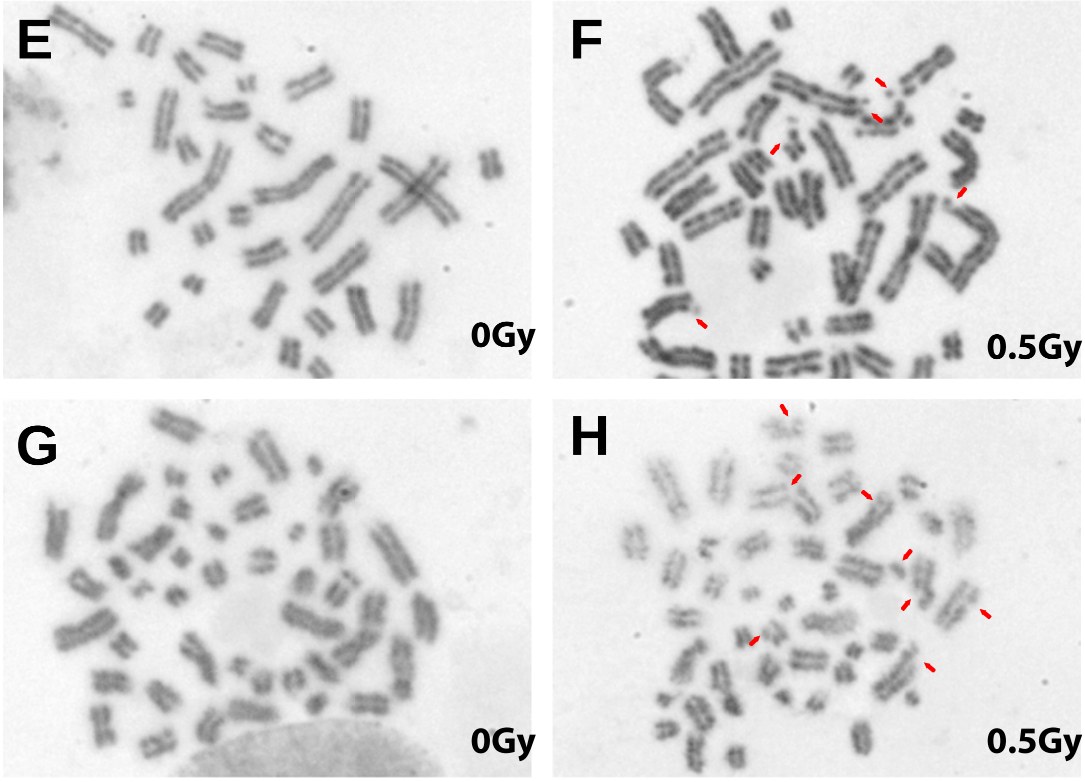


# Figure S12

## Supplementary Tables

### Table S1: siRNA sequences

| Target mRNA | siRNA sequence (5’ → 3’) | Provider |
|---|---|---|
| CAPH-1 | GGC-ACC-AGG-UUA-CUU-AAG-A55 | Eurogentec |
| CAPH-2 | ACA-CGC-AGA-UUA-CGG-AAC-A55 | Eurogentec |
| CAPH-3 | CGA-AGC-AGA-UCG-GAA-GUG-U55 | Eurogentec |
| CAPH-4 | CUU-UAG-GCC-UCG-ACG-CAA-A55 | Eurogentec |
| SMC2-1 | CAG-GUG-GUU-AUU-GGU-GGU-AGA-55 | Eurogentec |
| SMC2-2 | CUG-AAA-CGU-CGA-UAC-ACU-AUA-55 | Eurogentec |
| SMC2-3 | CAG-GUU-CGG-GCU-UCU-AAU-UUA-55 | Eurogentec |
| SMC2-4 | UUG-GAU-CUU-UCU-CAU-ACC-CAA-55 | Eurogentec |
| NCAPD3 | CAU-GGA-UCU-AUG-GAG-AGU-AUU-55 | Eurogentec |
| Negative control siRNA | UUC UCC GAA CGU GUC ACG UdTdT | Eurogentec |

### Table S2: Antibodies

| Antibody | Host/type | Dilution | Incubation time | Supplier |
|---|---|---|---|---|
| anti-mouse Alexa Fluor 488 | Goat/polyclonal | 1:400 (IF/FC) | 1 h | Invitrogen |
| anti-mouse Alexa Fluor 647 | Goat/polyclonal | 1:400 (IF) | 1 h | Invitrogen |
| anti-mouse IRDye 680LT | Goat/polyclonal | 1:10000 (WB) | 1 h | Li-COR Biosciences |
| anti-rabbit IRDye 680LT | Goat/polyclonal | 1:10000 (WB) | 1 h | Li-COR Biosciences |
| anti-rabbit IRDye 800LT | Goat/polyclonal | 1:1000 (WB) | 1 h | Li-COR Biosciences |
| ATR | Rabbit/polyclonal | 1:500 (WB) | overnight | Abcam plc |
| α-Tubulin | Mouse/monoclonal | 1:2000 (WB) | overnight | GeneTex |
| β-Actin | Rabbit/polyclonal | 1:30000 (WB) | overnight | Gene Tex |
| CAP-H | Rabbit/monoclonal | 1:1000 (WB) | overnight | Bethyl Laboratories |
| CAP-D3 | Rabbit/monoclonal | 1:2500 (WB) | overnight | Bethyl Laboratories |
| CtIP | Rabbit/monoclonal | 1:500 (WB) | overnight | Cell Signalling |
| GAPDH | Mouse/monoclonal | 1:20000 (WB) | overnight | UBP Bio |
| MRE11 | Mouse/monoclonal | 1:1000 (WB) | overnight | GeneTex |
| RPA70B | Mouse/monoclonal | 1:500 (IF/FC) | 1.5 h | IFMSB, UK Essen |

| | | | | |
|---|---|---|---|---|
| Rad51(14B4) | Mouse/monoclonal | 1:400 (IF/FC) | 1.5 h | Gene Tex |
| 53BP1 | Mouse/monoclonal | 1:500 (IF/FC) | 1.5 h | IFMSB, UK Essen |
| γ-H2AX | Mouse/monoclonal | 1:200 (IF/FC) | 1.5 h | Abcam plc |

**Table S3: Background of indicated foci in RPE-1 cell line**

| **γH2AX 0Gy** | **NC** | | | **siCAP-H+siCAP-D3** | | |
|---|---|---|---|---|---|---|
| | G1 | S | G2 | G1 | S | G2 |
| **1h** | 1.383 | 8.173 | 3.724 | 1.710 | 6.730 | 5.656 |
| **3h** | 1.533 | 6.553 | 3.833 | 1.840 | 8.276 | 3.903 |
| **6h** | 1.253 | 5.33 | 2.800 | 1.503 | 6.136 | 3.986 |

| **53bp1 0Gy** | **NC** | | | **siCAP-H+siCAP-D3** | | |
|---|---|---|---|---|---|---|
| | G1 | S | G2 | G1 | S | G2 |
| **1h** | 0.984 | 5.495 | 4.217 | 1.366 | 6.805 | 4.410 |
| **3h** | 0.832 | 6.112 | 3.688 | 1.375 | 4.678 | 3.906 |
| **6h** | 1.240 | 4.779 | 3.961 | 2.262 | 5.115 | 5.883 |

| **RAD51 0Gy** | **NC** | | | **siCAP-H+siCAP-D3** | | |
|---|---|---|---|---|---|---|
| | G1 | S | G2 | G1 | S | G2 |
| **1h** | 0.114 | 5.014 | 0.722 | 0.251 | 4.333 | 0.272 |
| **3h** | 0.378 | 4.569 | 0.857 | 0.057 | 5.192 | 0.828 |
| **6h** | 0.140 | 6.653 | 0.561 | 0.361 | 5.671 | 0.402 |

| **RPA70 0Gy** | **NC** | | | **siCAP-H+siCAP-D3** | | |
|---|---|---|---|---|---|---|
| | G1 | S | G2 | G1 | S | G2 |
| **1h** | 1.032 | 6.480 | 1.890 | 1.327 | 6.910 | 1.706 |

| | | | | | | |
|---|---|---|---|---|---|---|
| **3h** | 0.803 | 4.086 | 2.570 | 0.683 | 5.443 | 2.520 |
| **6h** | 0.513 | 6.030 | 1.240 | 0.840 | 4.163 | 1.227 |

**Table S4: Background of indicated foci in A549 cell line**

| **γH2AX 0Gy** | **NC** | | | **siCAP-H+siCAP-D3** | | |
|---|---|---|---|---|---|---|
| | G1 | S | G2 | G1 | S | G2 |
| **1h** | 1.426 | 10.350 | 5.387 | 1.440 | 11.426 | 4.48 |
| **3h** | 1.260 | 8.021 | 5.163 | 1.376 | 10.653 | 5.853 |
| **6h** | 2.293 | 7.657 | 3.586 | 0.967 | 7.86 | 3.403 |

| **53bp1 0Gy** | **NC** | | | **siCAP-H+siCAP-D3** | | |
|---|---|---|---|---|---|---|
| | G1 | S | G2 | G1 | S | G2 |
| **1h** | 1.118 | 3.887 | 2.126 | 1.526 | 5.363 | 6.686 |
| **3h** | 1.557 | 4.085 | 4.966 | 1.856 | 6.054 | 5.247 |
| **6h** | 1.336 | 4.514 | 3.572 | 1.675 | 3.719 | 2.734 |

| **RAD51 0Gy** | **NC** | | | **siCAP-H+siCAP-D3** | | |
|---|---|---|---|---|---|---|
| | G1 | S | G2 | G1 | S | G2 |
| **1h** | 0.097 | 5.483 | 1.010 | 0.137 | 7.960 | 1.293 |
| **3h** | 0.293 | 5.390 | 0.467 | 0.343 | 4.173 | 0.990 |
| **6h** | 0.970 | 4.866 | 0.900 | 0.690 | 5.347 | 1.383 |

| **RPA70 0Gy** | **NC** | | | **siCAP-H+siCAP-D3** | | |
|---|---|---|---|---|---|---|
| | G1 | S | G2 | G1 | S | G2 |
| **1h** | 0.137 | 4.752 | 0.277 | 1.062 | 5.290 | 2.224 |

| | | | | | | |
|---|---|---|---|---|---|---|
| **3h** | 0.683 | 7.163 | 1.148 | 1.906 | 8.23 | 1.278 |
| **6h** | 1.374 | 5.453 | 1.005 | 1.510 | 3.110 | 2.260 |

**Table S5：Gene names and the Assay IDs used in RT-qPCR.**

| Gene abbreviation | Gene names | Assay IDs | Supplier |
|---|---|---|---|
| **ACTB** | Actin beta | Hs01060665_g1 | Thermo Fischer Scientific |
| **NCAPH** | Non-SMC condensin I complex subunit H | Hs01010752_m1 | Thermo Fischer Scientific |
| **NCAPD3** | Non-SMC condensin II complex subunit D3 | Hs00293608_m1 | Thermo Fischer Scientific |
| **MRE11** | MRE11 homolog A, double-strand break repair nuclease | Hs00967437_m1 | Thermo Fischer Scientific |
| **RBBPB(CTIP)** | RB binding protein 8, endonuclease | Hs01090339_m1 | Thermo Fischer Scientific |
| **DNA2** | DNA replication helicase/ nuclease 2 | Hs01055240_m1 | Thermo Fischer Scientific |
| **EXO1** | Exonuclease 1 | Hs01116190_m1 | Thermo Fischer Scientific |
| **BLM** | Bloom syndrome RecQ like helicase | Hs00172060_m1 | Thermo Fischer Scientific |

| NAR-02494-Q-2025-MIQE ITEM TO CHECK | IMPORTANCE | CHECKLIST |
|---|---|---|
| **EXPERIMENTAL DESIGN** | | |
| Definition of experimental and control groups | **E** | |
| Number within each group | **E** | **checked** |
| Assay carried out by core lab or investigator's lab? | D | |
| Acknowledgement of authors' contributions | D | |
| **SAMPLE** | | |
| Description | **E** | checked |
| Volume/mass of sample processed | D | |
| Microdissection or macrodissection | **E** | Not applicable |
| Processing procedure | **E** | Not applicable |
| If frozen - how and how quickly? | **E** | Not applicable |
| If fixed - with what, how quickly? | **E** | Not applicable |
| Sample storage conditions and duration (especially for FFPE samples) | **E** | 20℃<br>RNA sample: -80℃ |
| **NUCLEIC ACID EXTRACTION** | | |
| Procedure and/or instrumentation | **E** | checked |
| Name of kit and details of any modifications | **E** | checked |
| Source of additional reagents used | D | |
| Details of DNase or RNAse treatment | **E** | checked |
| Contamination assessment (DNA or RNA) | **E** | |
| Nucleic acid quantification | **E** | checked |
| Instrument and method | **E** | checked |
| Purity (A260/A280) | D | |
| Yield | D | |
| RNA integrity method/instrument | **E** | checked |
| RIN/RQI or Cq of 3' and 5' transcripts | **E** | checked |
| Electrophoresis traces | D | |
| Inhibition testing (Cq dilutions, spike or other) | **E** | checked |
| **REVERSE TRANSCRIPTION** | | |
| Complete reaction conditions | **E** | checked |
| Amount of RNA and reaction volume | **E** | checked |
| Priming oligonucleotide (if using GSP) and concentration | **E** | Not applicable |
| Reverse transcriptase and concentration | **E** | checked |
| Temperature and time | **E** | checked |
| Manufacturer of reagents and catalogue numbers | D | |
| Cqs with and without RT | D* | checked |
| Storage conditions of cDNA | D | checked |
| **qPCR TARGET INFORMATION** | | |
| If multiplex, efficiency and LOD of each assay. | **E** | Not applicable |
| Sequence accession number | **E** | checked |
| Location of amplicon | D | |
| Amplicon length | **E** | checked |
| *In silico* specificity screen (BLAST, etc) | **E** | |
| Pseudogenes, retropseudogenes or other homologs? | D | |
| Sequence alignment | D | |
| Secondary structure analysis of amplicon | D | |
| Location of each primer by exon or intron (if applicable) | **E** | |
| What splice variants are targeted? | **E** | checked |
| **qPCR OLIGONUCLEOTIDES** | | |
| Primer sequences | **E** | checked |
| RTPrimerDB Identification Number | D | |
| Probe sequences | D** | |
| Location and identity of any modifications | **E** | checked |
| Manufacturer of oligonucleotides | D | |
| Purification method | D | |
| **qPCR PROTOCOL** | | |
| Complete reaction conditions | **E** | checked |
| Reaction volume and amount of cDNA/DNA | **E** | checked |
| Primer, (probe), Mg++ and dNTP concentrations | **E** | checked |
| Polymerase identity and concentration | **E** | checked |
| Buffer/kit identity and manufacturer | **E** | checked |
| Exact chemical constitution of the buffer | D | |
| Additives (SYBR Green I, DMSO, etc.) | **E** | checked |
| Manufacturer of plates/tubes and catalog number | D | |
| Complete thermocycling parameters | **E** | checked |
| Reaction setup (manual/robotic) | D | |
| Manufacturer of qPCR instrument | **E** | checked |
| **qPCR VALIDATION** | | |
| Evidence of optimisation (from gradients) | D | |
| Specificity (gel, sequence, melt, or digest) | **E** | checked |
| For SYBR Green I, Cq of the NTC | **E** | checked |
| Standard curves with slope and y-intercept | **E** | Not applicable |
| PCR efficiency calculated from slope | **E** | |
| Confidence interval for PCR efficiency or standard error | D | |
| r2 of standard curve | **E** | Not applicable |
| Linear dynamic range | **E** | Not applicable |
| Cq variation at lower limit | **E** | Not applicable |
| Confidence intervals throughout range | D | |
| Evidence for limit of detection | **E** | Not applicable |
| If multiplex, efficiency and LOD of each assay. | **E** | Not applicable |
| **DATA ANALYSIS** | | |
| qPCR analysis program (source, version) | **E** | checked |
| Cq method determination | **E** | checked |
| Outlier identification and disposition | **E** | checked |
| Results of NTCs | **E** | checked |
| Justification of number and choice of reference genes | **E** | checked |
| Description of normalisation method | **E** | checked |
| Number and concordance of biological replicates | D | |
| Number and stage (RT or qPCR) of technical replicates | **E** | checked |
| Repeatability (intra-assay variation) | E | checked |
| Reproducibility (inter-assay variation, %CV) | D | checked |
| Power analysis | D | |
| Statistical methods for result significance | **E** | checked |
| Software (source, version) | E | checked |
| Cq or raw data submission using RDML | **D** | |

**Table 1.** MIQE checklist for authors, reviewers and editors. All essential information (E) must be submitted with the manuscript. Desirable information (D) should be submitted if available. If using primers obtained from RTPrimerDB, information on qPCR target, oligonucleotides, protocols and validation is available from that source.

*: Assessing the absence of DNA using a no RT assay is essential when first extracting RNA. Once the sample has been validated as RDNA-free, inclusion of a no-RT control is desirable, but no longer essential.

**: Disclosure of the probe sequence is highly desirable and strongly encouraged. However, since not all commercial pre-designed assay vendors provide this information, it cannot be an essential requirement. Use of such assays is advised against.
